# The interaction between oxidative stress and Schwann cells

**DOI:** 10.3389/ebm.2026.11146

**Published:** 2026-08-27

**Authors:** Sihan Yang, Zijun Zhang, Qing Liao, Qipeng Ouyang, Yongyou Ye, Wei Lu, Zhendong Jiang

**Affiliations:** 1 Department of Basic Education, Zhuhai Campus of Zunyi Medical University, Zhuhai, China; 2 Xiaolan People’s Hospital of Zhongshan, Zhongshan, China; 3 College of Basic Medicine, Guangdong Jiangmen Chinese Medical College, Jiangmen, China

**Keywords:** oxidative stress, reactive oxygen species, Schwann cells, peripheral nerve injury, nerve guidance conduit, nerve tissue engineering, antioxidation, mitophagy

## Abstract

After peripheral nerve injury (PNI), a moderate increase in reactive oxygen species (ROS) levels can promote the proliferation and differentiation of Schwann cells (SCs) and participate in signal transduction. However, excessive ROS can trigger oxidative stress, and its harmful effects can overshadow the beneficial ones, such as inducing apoptosis, activating inflammation, disrupting environmental stability, and severely hindering neural processes. Therefore, if the damage caused by oxidative stress to SCs can be effectively alleviated, it will provide a new approach for the precise regeneration between peripheral nerves and target organs, thereby reducing the complications of PNI. With the advancements in materials science and neural tissue engineering, functional and precisely designed neural scaffolds and conduits have emerged as highly promising therapeutic strategies. This article focuses on revealing the interaction between SCs and oxidative stress during nerve injury, as well as the innovative tissue engineering technologies and new paradigms for damage repair that have emerged based on this interaction. It aims to provide effective new methods for addressing temporary or permanent functional impairments of PNI/peripheral neuropathies.

## Impact statement

After peripheral nerve injury (PNI), a moderate increase in reactive oxygen species (ROS) levels can promote the proliferation and differentiation of Schwann cells (SCs) and participate in signal transduction. However, excessive ROS can trigger oxidative stress, and its harmful effects can overshadow the beneficial ones, such as inducing apoptosis, activating inflammation, disrupting environmental stability, and severely hindering neural processes. Therefore, if the damage caused by oxidative stress to SCs can be effectively alleviated, it will provide a new approach for the precise regeneration between peripheral nerves and target organs, thereby reducing the complications of PNI. This review provides a comprehensive overview of the mechanisms of excessive ROS production, oxidative stress-induced SCs’ damage, and endogenous antioxidant defense in PNI, as well as emerging nerve tissue engineering strategies for intervention. By constructing a discussion framework that progresses from “mechanism elucidation” to “technology integration” and ultimately to “clinical translation,” this work strives to provide guidance for the future development of intelligent neural repair systems that possess both dynamic responsiveness and functional remodeling capabilities.

## Introduction

Peripheral nerve injury (PNI), a highly prevalent neurological disease, is often caused by trauma, traffic accidents, mechanical tension, and other factors, and has become a heavy burden on global public health and economic development [1]. Despite the regenerative potential of peripheral nerves, their slow regeneration rate may lead to dysfunction or irreversible damage of target organs due to long-term loss of innervation, such as dyskinesia, sensory abnormalities, pain sensitization, and muscular atrophy, and some patients may even face lifelong disability risk [2]. The primary challenge for PNI repair is the disruption of the microenvironment triggered by oxidative stress (OS) [3]. Reactive oxygen species (ROS), originating from oxygen metabolism, can harm cells by interacting with biological macromolecules such as lipids, proteins, and nucleic acids. Its excessive accumulation has been confirmed to cause in-cell and tissue damage. During aging, it is the leading contributor to cell and tissue damage [4, 5]. Under physiological conditions, the endogenous intracellular antioxidant system can maintain an exquisite metabolic balance between ROS generation and scavenging through sophisticated regulation [6, 7].

However, during the repair process of PNI, combined cellular damage and inflammation exacerbate mitochondrial electron transport chain (ETC.) leakage, further stimulating ROS production. Such as NADPH oxidase (Nox), oxidative burst during ischemia-reperfusion injury (IRI), and so forth. Nox, oxidative burst during Ischemia-Reperfusion Injury (IRI), and other ROS generation pathways. Excessive ROS production overwhelms endogenous antioxidant defenses, disrupting the critical balance between oxidative stress and reduction [8, 9]. The unbalanced oxidative microenvironment can not only directly induce neuronal apoptosis but also facilitates SC senescence transition, which disrupts the regenerative microenvironment and diminishes neural repair capacity [4, 10, 11]. As crucial regulators of peripheral nerve regeneration, Schwann cells (SCs) modulate the redox microenvironment through antioxidant mechanisms and signaling pathways (e.g., Hedgehog), which may help preserve neuronal redox homeostasis and support regenerative responses [12, 13]. Of interest, ROS fluctuations serve dual regulatory functions, mediating both host immune responses and essential physiological signaling pathways: immune cells activate the inflammatory response by synergistically releasing large amounts of ROS to clear pathogens, whereas low concentrations of ROS act as redox signaling molecules that participate in the regulation of cell proliferation, differentiation, and other basic life activities [14, 15]. Moreover, the interaction between OS and SCs in neuropathies such as diabetic peripheral neuropathy (DPN) and neurofibromatosis type 1 (NF1) also plays a key role in the pathological microenvironment [16, 17]. This duality of biological properties suggests the need for an in-depth analysis of the spatiotemporal distribution of ROS, the mechanisms of SC damage, and the dynamic regulation of antioxidant pathways. Such an analysis will provide a theoretical foundation for developing smart neural scaffolds with both anti-inflammatory and antioxidant functions.

Conventional neural scaffolds are single-function designs, challenging to respond to the complex dynamic changes in the regenerative microenvironment due to their single-function design, leading to deficient nerve fiber regeneration and constrained functional recuperation [18, 19]. The convergence of cell biology and advanced materials science has propelled neural tissue engineering beyond structural imitation, ushering in an era of functionally integrated regenerative strategies. This paradigm focuses on fabricating tripartite functional constructs that combine living cells, scaffold matrices, and bioactive signaling factors in three-dimensional architectures. Based on this approach, nerve guidance conduits (NGCs) are designed to actively direct axon growth by integrating SCs, neurotrophic factors, and other key regenerative components [20]. In addition, advanced NGC iterations feature embedded biosensing modules and responsive drug release mechanisms, allowing dynamic tracking of redox status and demand-based neuroprotection [21]. This dynamic regulatory mechanism overcomes the static limitations of traditional scaffolds. By precisely maintaining redox homeostasis, it simultaneously promotes vascular regeneration and the reconstruction of the bioelectrical signaling network [22, 23].

**FIGURE 1:**
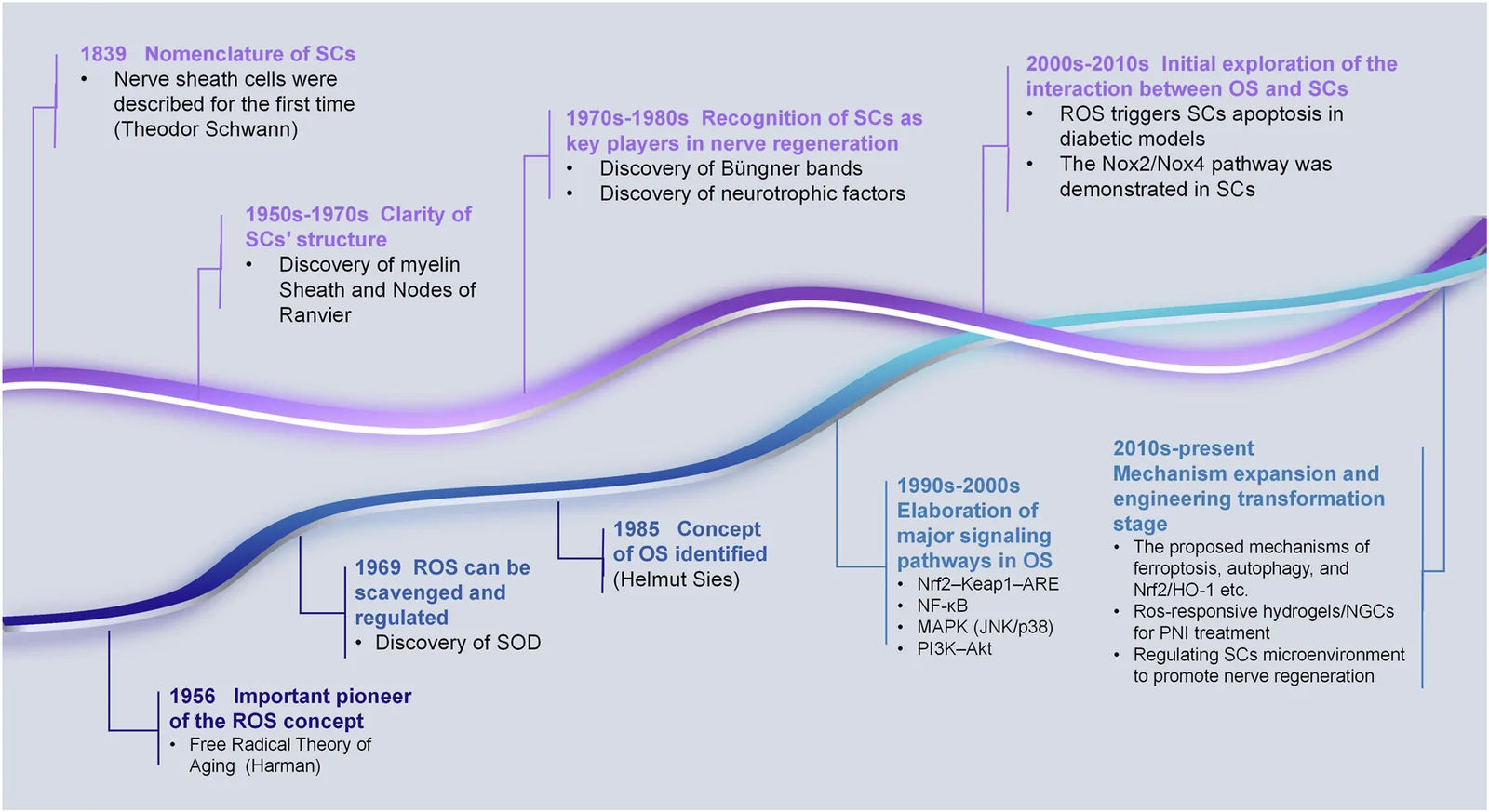
Timeline: The significant events of SC and OS research and the new paradigm of their interaction. Abbreviations: SCs, Schwann cells; OS, oxidative stress; ROS, reactive oxygen species; Nox2/Nox 4, NADPH Oxidase 2/NADPH Oxidase 4; NGCs, nerve guidance conduits; PI3K-Akt Pathway, Phosphatidylinositol 3-Kinase; p38, p38 Mitogen-Activated Protein Kinase; JNK, c-Jun N-terminal Kinase; MAPK, Mitogen-Activated Protein Kinase; Nrf2-Keap1-ARE, Nuclear Factor Kappa-Light-Chain-Enhancer of activated B cells.

In the future, with the development of ROS-responsive drug delivery systems, engineered exosome platforms, and advanced technologies such as 4D bioprinting, the analysis of the spatiotemporal dynamics of OS will drive breakthroughs in personalized treatment strategies. These advances are expected to support the sustained functional regeneration of complex nerve injuries. A summary timeline of significant events in SC and OS research is shown in Figure 1.

This review is built on a central framework: the dynamic interplay between OS and SCs dictates the success of peripheral nerve repair, and understanding this mechanism provides the blueprint for next-generation interventions. To strengthen the narrative flow, we organize our discussion into a structured progression: (1) Mechanism elucidation: uncovering how ROS production and endogenous antioxidant defenses govern SC fate; (2) Technology integration: highlighting how novel nerve tissue engineering, such as smart drug delivery and 4D bioprinting, actively intervenes in this OS-SC axis; (3) Clinical translation: evaluating these strategies in disease models and clinical applications. This structured approach aims to guide the evolution of neural repair from passive scaffolds to active, microenvironment-responsive systems.

## Retrieval strategy

**FIGURE 2:**
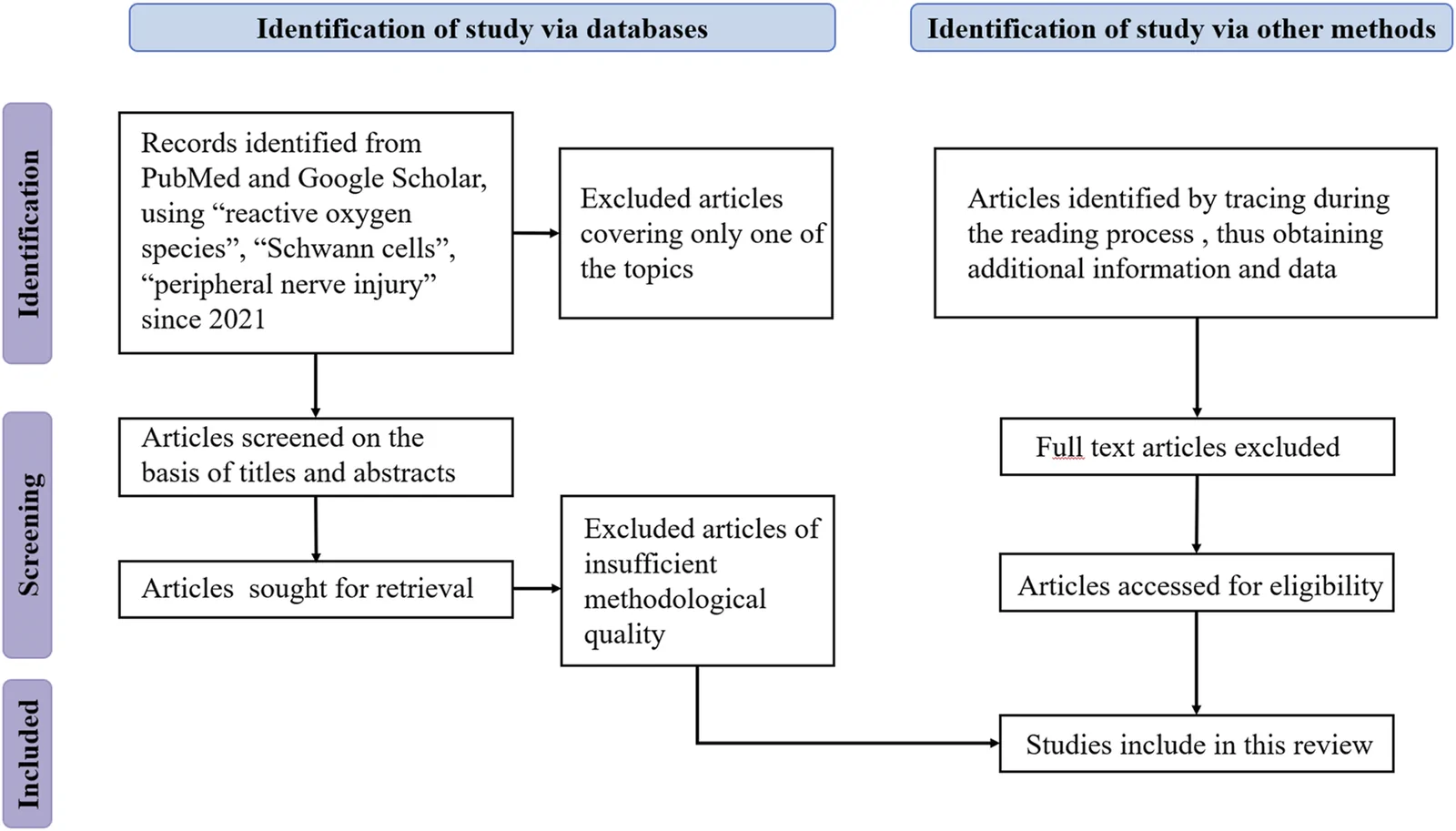
Flowchart of the retrieval process: This figure illustrates the strategy used in this review for searching the literature.

This study systematically retrieved literature from PubMed using combined keywords related to oxidative stress, reactive oxygen species, peripheral nerve injury, nerve tissue engineering, and nerve guidance conduits, covering the period from March 2021 to March 2026 without language or study type restrictions. To deeply explore the interplay between OS and PNI repair, articles focusing unidirectionally on either OS or PNI without addressing their interaction in nerve regeneration were excluded. Eligible high-quality studies underwent full-text assessment, and a snowballing approach was applied to reference lists for additional relevant publications. All searches were completed by June 2026, with the literature screening process detailed in Figure 2.

## The molecular mechanism of the interaction between SCs and oxidative stress

As a key response mechanism for the body to cope with harmful stimuli in the internal and external environment, OS manifests through the pathological accumulation of ROS and reactive nitrogen species (RNS), disrupting redox homeostasis [24]. ROS include strong oxidizing substances such as superoxide anion (O^2-^), hydroxyl radical (OH^−-^), and hydrogen peroxide (H_2_O_2_). Similarly, RNS encompasses highly reactive molecules like nitric oxide (NO) and peroxynitrite (ONOO^−^) [25]. These free radicals induce DNA strand breaks, protein conformational changes, and lipid peroxidation through direct oxidative damage or cascade reactions, ultimately leading to cellular dysfunction and compromised tissue regeneration.

This pathological microenvironment not only promotes neuronal apoptosis but also exacerbates the neuroregenerative microenvironment by inducing senescence-like phenotypic switching in SCs [4, 10, 11]. What is more noteworthy is that excessive accumulation of ROS can cause neuronal cells and SCs to arrest in the G0/G1 phase and enter a quiescent, senescent, or even apoptotic state. This condition significantly impairs their core repair functions, including myelin clearance, neurotrophic factor secretion, and axon regeneration. As a result, the overall regenerative capacity of the nerves is compromised [26, 27].

Furthermore, recent studies have also indicated that ROS/RNS can modify key signaling proteins such as components of the Mitogen-Activated (MAPK), Phosphoinositide 3-Kinase/Protein kinase B (PI3K/Akt), and Nuclear Factor Erythoid 2-Related Factoe 2 (Nrf2) pathways, influencing the dedifferentiation and redifferentiation processes of SCs [28–30]. They also participate in regulating the immune system and metabolic reprogramming of the regenerative microenvironment, forming a complex network of interactions between OS and SCs.

### The generation of ROS and its interaction with SCs

#### Mitochondrial electron leakage and calcium overload

Under physiological conditions, as the major cellular ROS producer, the ETC generates free radicals through unavoidable electron escape during adenosine triphosphate (ATP) synthesis. These leaked electrons readily react with molecular oxygen, generating ROS species, such as O^2-^. Mitochondrial-derived ROS represent one of the primary pathogenic mediators in OS development due to their continuous production during aerobic respiration [31]. Its initial site of generation is usually considered to be the semiquinone radical (QH^•^) or reduced flavin (FMN and FAD). At mitochondrial respiratory Complex I (CI), O^2-^ may be generated at either the flavin or I_Q_ site and partitions completely into the mitochondrial matrix. In mitochondrial respiratory Complex III (CIII), O^2-^ is produced at the Qo site and released into the membrane space and matrix [32]. The vast majority of physiological ROS originates from the electron leakage at CI and CIII, and a small fraction arises from the direct electron leakage from the 3Fe-4S cluster of mitochondrial respiratory Complex II (CII) to oxygen, yielding O^2-^ [33]. At physiological concentrations, appropriate amounts of ROS are essential factors involved in SCs signaling and metabolic regulation.

Moreover, in pathological niches with intact glia-neuron units, drastic microenvironmental changes trigger significant Ca^2+^ influx through activation of SC surface channels such as TRPA1. This aberrant Ca^2+^ accumulation serves as the priming factor for subsequent mitochondrial dysfunction [34, 35]. Mechanistically, Kievit et al. observed in an *in vitro* axotomy model that mitochondrial ROS production preceded late-stage Ca^2+^ entry [36]. Owever, this sequence likely reflects a distinctive phenotype of glia-free isolated axon systems. In this highly simplified model, severed axons lack physical ensheathment and metabolic support from SCs. They undergo rapid, autonomous NAD^+^ depletion, which triggers intrinsic mitochondrial collapse and places mitochondrial ROS generation upstream of channel activation. Consequently, in in vivo, *ex vivo*, or co-culture systems with preserved SC-axon communication, extrinsic glia-derived oxidative stress and localized Ca^2+^ perturbations typically dominate the induction of mitochondrial dysfunction.

#### Nox activation and ROS generation

The Nox family remains as enzymes that specialize in ROS generation and still play a significant role, with members including NOX1, NOX2 (gp91phox), NOX3, NOX4, NOX5, and DUOX1 and DUOX2 [37]. The distribution of members of this family varies in the peripheral nervous system: dorsal root ganglion neurons express Nox1, Nox2, and Nox4, macrophages express Nox2 and Nox4, and SCs express Nox1 and Nox4 [38]. Due to the structural homology of Nox enzymes, the core mechanism of ROS production is basically the same. This reactive species subsequently undergoes dismutation, either spontaneously or via superoxide dismutase (SOD) catalysis, yielding H_2_O_2_. The subsequent fate of H_2_O_2_ is highly dependent on the local microenvironment. H_2_O_2_ undergoes the Fenton reaction catalyzed by transition metal ions, involving Fe^2+^, generating OH^−^. In the absence of such ions, residual H_2_O_2_ serves as a substrate for myeloperoxidase (MPO) to generate hypochlorous acid (HOCl), a potent oxidant involved in innate immune responses [39].

Distinct activation modes represent a key factor underlying the functional diversity among enzymes of the Nox family [38]. Regarding Nox1 expressed in SCs, De Logu et al. demonstrated that ROS derived from macrophage Nox2 activate Transient Receptor Potential Ankyrin 1 (TRPA1) channels in SCs, leading to calcium influx and subsequent activation of Nox1. The oxidative product released by activated Nox1 is H_2_O_2_. Additionally, a high-glucose environment serves as a key activator of Nox4 in Schwann cells. Elevated blood glucose levels inhibit the liver X receptor (LXR) pathway in macrophages, thereby relieving its transcriptional repression on Nox4. This approach leads to upregulated Nox4 expression and subsequently results in massive ROS production [40].

#### IRI-induced oxidative bursts

**FIGURE 3:**
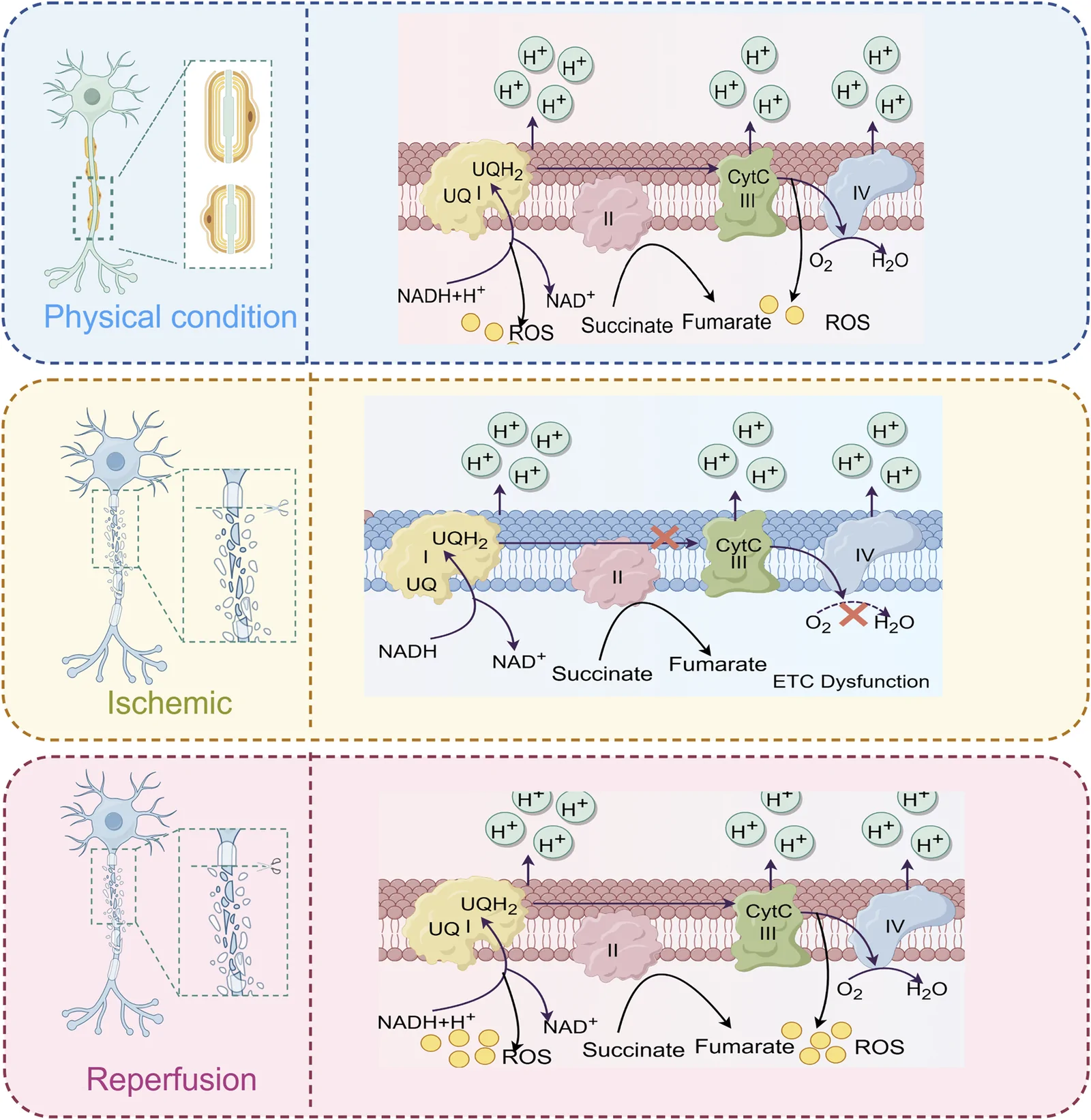
Under physiological conditions, a small amount of ROS leaks from CI and CIII during normal electron transfer in the respiratory chain. However, PNI-induced ischemia and hypoxia impair the synthesis of H_2_O at CIV, leading to electron transfer blockage and the accumulation of succinate at CII. Upon reperfusion, as the respiratory chain resumes function, the rapid oxidation of accumulated succinate generates excessive electrons, which flow in reverse to CI via RET, resulting in a burst of ROS production at CI.

IRI is a biphasic pathological process comprising ischemic and reperfusion phases [37, 41]. At the ischemic stage, blockage of oxidative phosphorylation in mitochondria results in the cessation of ATP synthesis, ionic homeostasis imbalance, calcium overload, intracellular acidosis, and accumulation of succinate [42]. Among these changes, succinate is a mitochondrial metabolite, and evidence suggests that the tissue-specific retention of this molecule serves as a metabolic signature of ischemia, while also functioning as a critical mediator of reperfusion-induced oxidative damage. Under normal physiological conditions, the proton drive (Δp) is less than the redox drive (ΔEH) to maintain the membrane potential (ΔΨm) required for ATP synthesis. However, metabolic alterations during hypoxia invert the relationship (Δp > ΔEH), and this is further exacerbated during reperfusion. Then the abrupt rise in oxygen availability during reperfusion facilitates the rapid oxidation of accumulated succinate by succinate dehydrogenase. This process leads to a burst of electron flow into the mitochondrial respiratory chain, resulting in a marked depletion of the reduced coenzyme Q pool. These changes ultimately initiate Reverse Electron Transport (RET), in which electrons from ubiquinol are reversed and transferred back to CI, and RET triggers a burst of ROS during reperfusion at CI [43, 44]. RET acts as a key amplifier of OS effects during reperfusion, perpetuating a self-reinforcing vicious cycle that exacerbates oxidative damage (Figure 3).

Despite the tissue-specificity of OS mechanisms in different tissues (e.g., neural, cardiac, hepatic, renal), the highly conserved structure of the mitochondrial ETC makes theoretical migration between tissues possible [45]. Moreover, there is evidence that ischemic reperfusion injury secondary to PNI induces an oxidative burst [46, 47]. However, the initiation sites and regulatory mechanisms of ROS generation following RET remain poorly characterized, with limited studies available. In contrast, numerous studies based on myocardial ischemia–reperfusion models have elucidated the mechanism of RET and identified complex I as the primary site of RET-induced ROS production [48]. Thus, these findings in cardiac research may serve as a valuable reference for exploring similar mechanisms in SCs models. Based on this, they can seek clues to formulate hypotheses that are applicable to the PNI model and test them experimentally. This approach could facilitate fundamental exploration at the level of OS mechanisms.

### Direct and indirect damage mechanisms of OS on SCs

#### *Oxidative damage* and apoptosis in SCs

In the OS state, polyunsaturated fatty acids (PUFAs) on the SC membrane are the primary targets of ROS damage. Among these, the essential fatty acids n-6 and n-3 are required for standard membrane structure and fluidity as well as for arachidonic acid-like production. Not only do ROS directly attack PUFAs, leading to disruption of cell membrane integrity, but lipid peroxides generated by this process also cause neurological changes such as axonopathy and demyelination when accumulated over time [49]. Moreover, as PUFAs are ubiquitously present in the phospholipid bilayer of organelle membranes, ROS can also damage the subcellular structures of SCs, such as altered mitochondrial membrane permeability and endoplasmic reticulum stress.

Crucially, the progression from general oxidative damage to ferroptosis in SCs is tightly linked to the collapse of the endogenous antioxidant defense system under sustained OS [50]. Excessive ROS generation rapidly depletes intracellular glutathione (GSH), which subsequently inactivates Glutathione Peroxidase 4 (GPX4). As GPX4 is the primary enzyme responsible for reducing toxic lipid hydroperoxides to non-toxic lipid alcohols, its OS-induced inactivation removes the critical brake on lipid peroxidation [51]. Furthermore, severe OS can impair System Xc- (SLC7A11), restricting cystine uptake and exacerbating GSH depletion, thereby forming a vicious cycle that sensitizes SCs to ferroptosis [52].

**FIGURE 4:**
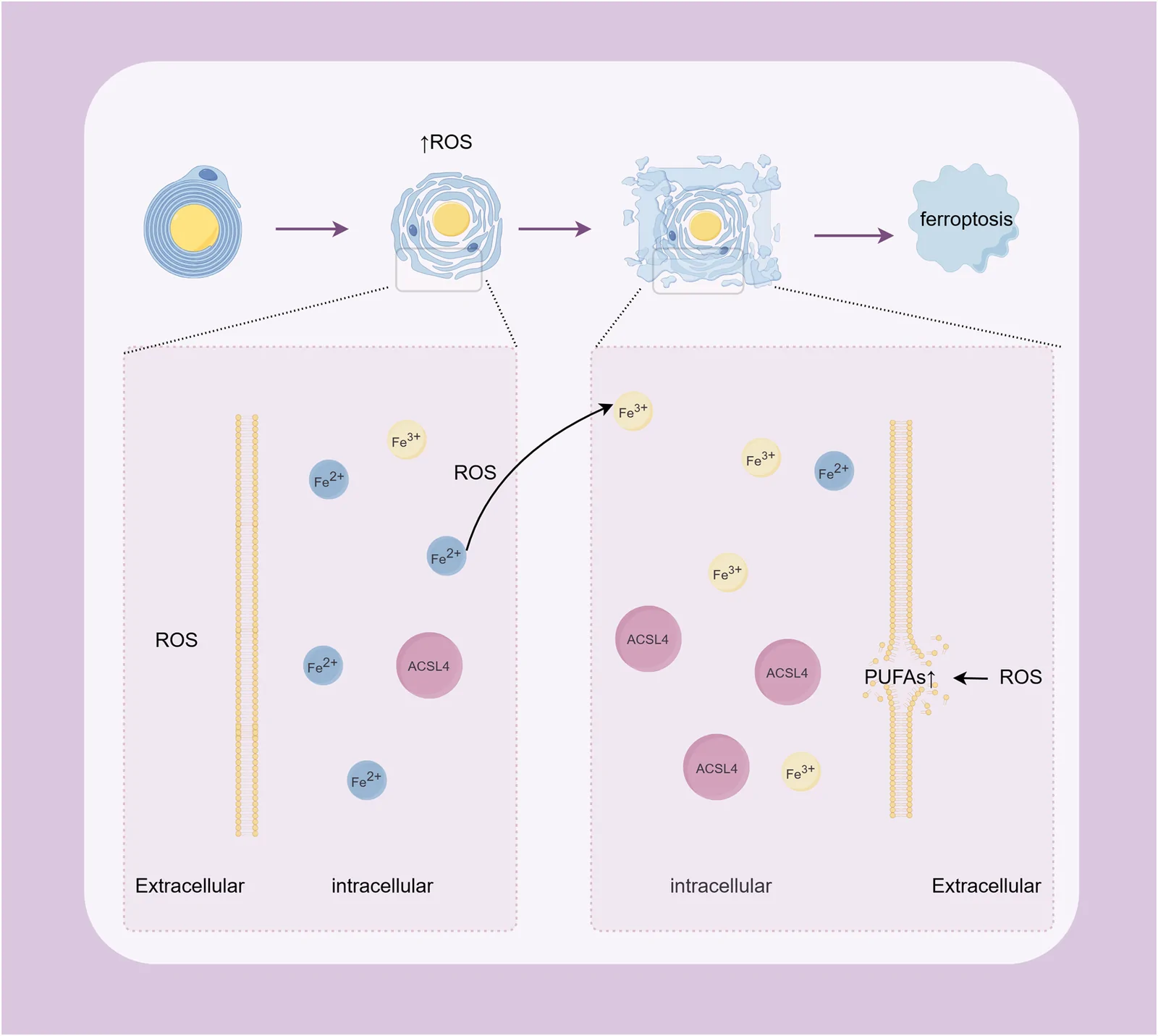
The mechanism of ferroptosis induced by oxidative stress in Schwann cells.

In addition, the Fenton reaction triggered by excessive H_2_O_2_ and ferrous ions (Fe^2+^), in SCs would induce ferroptosis [53]. Iron death is an iron-dependent form of non-apoptotic cell death, and the response is mediated by increased intracellular iron content and Acyl-CoA Synthetase Long-Chain Family Member 4 (ACSL4) expression. Divalent iron catalyzes the Fenton reaction, initiating lipid peroxidation that disrupts and ruptures the SC membrane, ultimately resulting in cell death. ACSL4 exacerbates the damage to the cell membrane by increasing the content of polyunsaturated fatty acids in the cell membrane, making the lipid peroxidation reaction more prone to occur. In contrast, knockdown of the ACSL4 gene significantly suppresses lipid peroxidation and iron-mediated cytotoxicity, while the morphology and function of the cell membrane are better maintained [54] (Figure 4). These results underscore the critical role of ROS in mediating ferroptosis in SCs and highlight the targeted inhibition of ferroptosis as a highly promising therapeutic strategy for mitigating OS-induced damage and enhancing SC survival.

In the context of peripheral neuropathy and nerve regeneration, OS-induced SC ferroptosis has profound pathological consequences. It not only directly leads to demyelination [55], but also disrupts local iron homeostasis and releases lipid peroxides into the microenvironment [56]. This toxic milieu exacerbates neuroinflammation and impedes macrophage-mediated myelin debris clearance, ultimately creating a hostile environment that severely hinders axonal regrowth and subsequent remyelination [56]. These results underscore the critical role of ROS in mediating SC ferroptosis. They also highlight targeted ferroptosis inhibition as a highly promising therapeutic strategy. This approach could mitigate OS-induced damage while enhancing SC survival and overall nerve regeneration [57].

#### Inhibitory effects of OS on SC function

**FIGURE 5:**
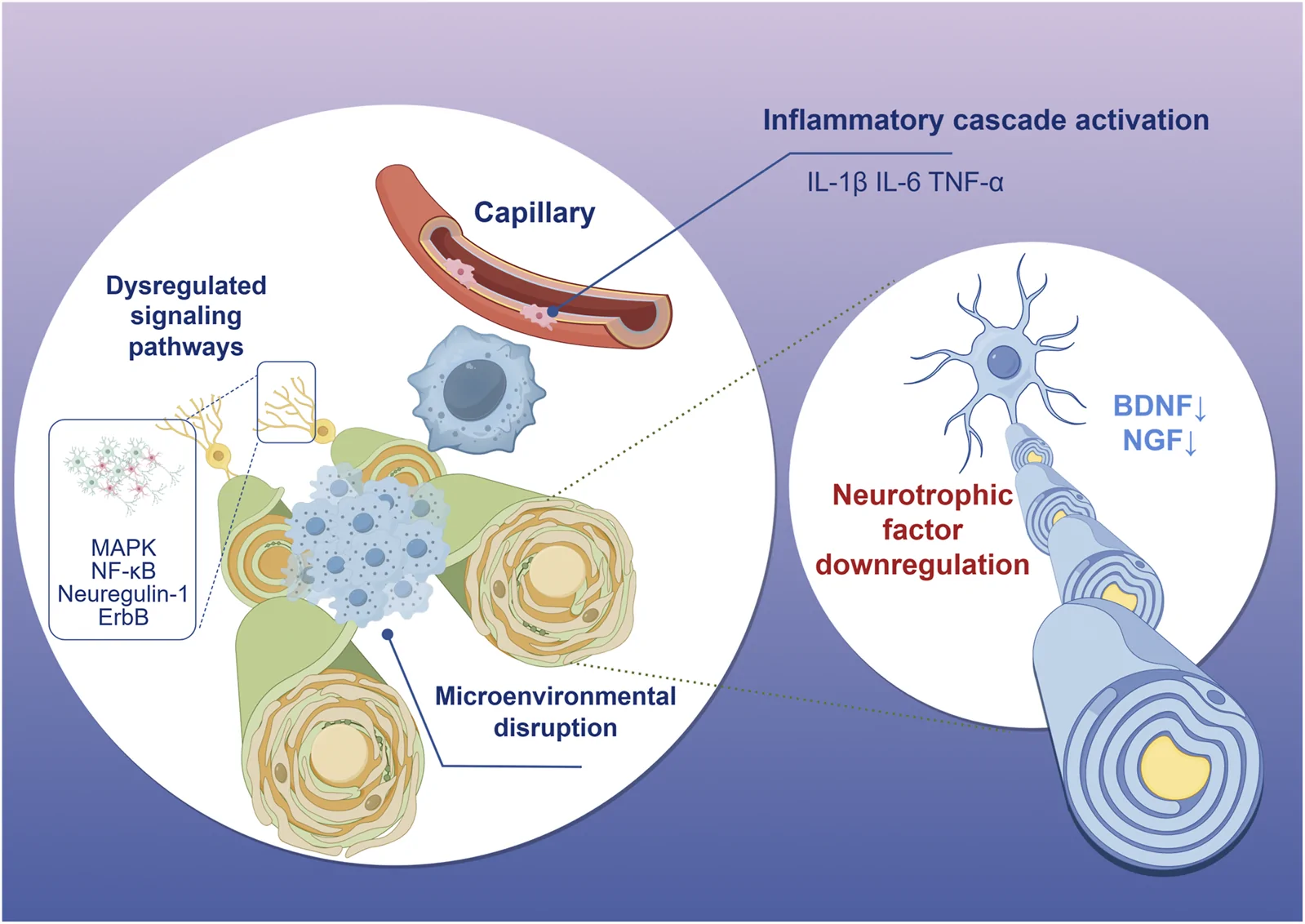
OS indirectly impairs the repair function of SCs through disrupting the neural microenvironment and the signal communication between cells, mainly in four aspects: (1) dysregulation of key signaling pathways; (2) destruction of ECM; (3) activation of inflammatory cascade reactions; (4) inhibition of neurotrophic factor secretion. These processes are interrelated and collectively form a vicious cycle that inhibits neural regeneration.

SCs can remove myelin sheaths, guide axonal regeneration, and provide the physical structural and trophic support that will, in turn, promote Neural repair of peripheral nerves [58]. However, OS directly or indirectly impairs the repair capacity of Schwann cells through a multidimensional mechanism that impairs the following: (1) Proliferation limitation: OS significantly inhibits the proliferation and survival of SCs, and its effects are dose-dependent but do not lead to a complete arrest of SCs’ proliferation in the concentration range studied [54, 59]. (2) Inhibition of neurotrophic factor secretion: Brain-derived neurotrophic factor (BDNF) and nerve growth factor (NGF) can promote the migration of SC [60]. In Ma et al.'s experiments, H2O2-induced OS significantly decreased the levels of BDNF and NGF secreted by SCs [61]. (3) Extracellular matrix alterations: the extracellular matrix (ECM) regulates SCs’ proliferation, migration, and myelin formation [62]. It has been demonstrated in spinal cord injury studies that excess ROS can cause degradation of key scaffolding components, such as laminin and fibronectin, in the extracellular matrix ECM, destroying the physical support structures necessary for axonal regeneration [63]. Peripheral nerve tissue is also rich in similar ECM components, such as laminin and fibronectin [62]. Therefore, OS may similarly damage this ECM and impair nerve regeneration. In addition, the OS conditions in peripheral nerve tissues contributed to the dysfunction of mitochondrial metabolism, immunomodulation, and other functions in SCs [64, 65]. If SCs are in a high-glycemic environment, OS will synergistically exacerbate the metabolic disturbances of the cells [66]. The above interactions between the pathologic microenvironment and SCs will be further developed in subsequent sections (Figure 5).

### Dynamic balance and regulatory mechanism of ROS content

#### The dual role of ROS and stage-specific regulation

ROS function as pivotal signaling mediators in the context of cellular injury, orchestrating adaptive responses, damage resolution, autophagic homeostasis, and intracellular signal transduction [67]. Such regulatory roles of ROS are particularly prominent in the repair process following PNI. Upon activation of the repair program, SCs undergo a series of coordinated stages, including dedifferentiation, microenvironmental remodeling, the secretion of neurotrophic factors, and axonal regeneration. Each stage critically depends on the dynamic balance between ROS generation and elimination.

It is worth noting that ROS are not merely harmful metabolic byproducts. Recent studies have demonstrated that injury-induced H_2_O_2_ acts as a pro-regenerative signal. Negro et al. showed that H_2_O_2_ activates ERK1/2 and c-Jun to drive Schwann cell phenotype conversion. Simultaneously, it upregulates Ctgf via YAP/TEAD to remodel the ECM, which guides Schwann cell migration and oriented axonal growth [68, 69]. Moreover, ROS exhibit stage-specific regulatory effects during nerve repair, driven by a concentration window phenomenon. At certain phases, maintaining an appropriate level of ROS can positively influence SC behavior. This underscores the dose-dependent and temporally specific nature of ROS signaling in the SC-mediated repair process. For instance, during the dedifferentiation phase, SCs may increase ROS production to activate pro-proliferative signaling pathways, thereby facilitating adaptive cellular responses and promoting proliferation [70]. While moderate levels of ROS facilitate intracellular signaling and cytoskeletal remodeling during the axon guidance stage, they also contribute to growth cone navigation. Furthermore, ROS participates in bidirectional interactions with the autophagy system via the mTOR pathway. This interplay helps sustain the metabolic homeostasis of SCs [71].

In summary, the generation and elimination of intracellular ROS is a tightly regulated process, and the maintenance of redox equilibrium is essential for preserving normal cellular physiology. Consequently, dynamic and accurate regulation of intracellular ROS levels across different stages of nerve injury and repair is a crucial determinant of SCs’ repair capacity and successful peripheral nerve regeneration [72–74].

#### Equilibrium of pro-survival and pro-apoptotic pathways

At the molecular level, this precise regulation relies heavily on the dynamic equilibrium between two core signaling networks: pro-survival and pro-apoptotic pathways [57]. Excessive *in vivo* ROS can directly or indirectly activate multiple intracellular signaling pathways, influencing cell fate by regulating gene expression. In pro-survival signaling, ROS promote cell survival by triggering pathways such as Nrf2/ARE, MAPK/ERK, PI3K/Akt, and NF-κB [29, 75–77], which upregulate the expression of molecular chaperones. Conversely, in pro-apoptotic signaling, elevated ROS activate the JAK/STAT, ATM/p53, and MAPK/JNK/p38 pathways [46], enhancing the expression of pro-apoptotic proteins such as Bax and Caspase-3, ultimately leading to cell apoptosis. Therefore, achieving a dynamic balance between these opposing pathways through the precise regulation of key signaling nodes represents a central mechanism for governing SC survival.

This molecular foundation enables the manipulation of ROS kinetics through engineering strategies. For example, mung bean-derived carbon dots (MB-CDs) activate Nrf2 and downstream Heme Oxygenase-1 (HO-1) and GPX4, converting hemoglobin into CO, Fe^2+^, and bilirubin to halt SC ferroptosis via lipid peroxidation inhibition [30]. Similarly, porous nanocomposites effectively activate the PI3K/Akt signaling pathway through the sustained release of selenium, reducing ROS generation while suppressing apoptosis, and endowing cells with enhanced migration and nerve regeneration capabilities [75].

These mechanisms construct a systematic framework for ROS regulation: applying active substances restores intracellular anti-oxidation and anti-apoptosis homeostasis, while biocompatible slow-release materials, such as nanoplatforms or hydrogels, enhance spatiotemporal targeting during nerve regeneration. Integrating fragmented pathway research into a synergistic oxidative stress network deepens the understanding of ROS dynamics and provides a solid theoretical foundation [76, 77] for coupling molecular mechanisms with physical stimuli, such as biomimetic matrix topographies, to strategically tailor therapeutic intracellular responses.

#### Methodologies and challenges in ROS detection

Understanding and precise monitoring of this dynamic balance is a prerequisite for intervening in the ROS-SC interaction. Nevertheless, molecular characteristics such as millisecond half-life and complex diffusion kinetics pose a significant challenge for its accurate detection. Currently, commonly employed methods for ROS detection include electron paramagnetic resonance (EPR), fluorescent staining, chemiluminescence, chromatographic techniques, spectrophotometry, electrochemical biosensors, and fluorescent protein-based approaches. However, these mainstream detection techniques still exhibit various limitations [78]. To facilitate a critical assessment of these methods, we have synthesized their key advantages and limitations in Table 1, providing a comprehensive perspective on their utility for monitoring ROS dynamics in nerve regeneration research.

**TABLE 1:** Comparison of mainstream ROS detection technologies in terms of sensitivity, dynamics, specificity, and *in vivo* applicability.

ROS detection technologies	Sensitivity	Temporal & spatial dynamics	Specificity	Applicability	References
EPR	Irreversible cumulative endpoint detection	Bulk measurements lacking subcellular resolution	Capable of targeting specific ROS species	ROS capture strictly *in vivo* (via injection/incubation) with *ex vivo* readouts	[79–81]
Fluorescent staining	Limited response to low ROS; DCFH-DA requires peroxidases or metal ions for H_2_O_2_; Nonspecific oxidation and redox cycling amplify signals, causing artifacts	Irreversible end-point accumulation; Conventional probes lack real-time dynamic tracking; Advanced probes enable organelle targeting but not traditional ones	Poor specificity; DCFH-DA oxidized by ·OH, ONOO^−^, heme proteins, glutathione radicals, *etc.*,; DHE/MitoSOX products have overlapping fluorescence spectra	Suitable for cells, tissue sections, and transparent models; Deep-tissue *in vivo* imaging limited by light penetration and autofluorescence	[82–84]
Electrochemical sensors	µM to nM detection limits; Material- and probe-dependent; tunable via design	Real-time, *in situ*, dynamic; µm-scale resolution via microelectrodes implanted in brain regions; rapid response; No sub-organellar resolution	Limited intrinsic selectivity; Achieved via probe/receptor design to exclude electroactive interferents	*In vitro* cell supernatants; *in vivo* brain monitoring; Implantable long-term potential; microtrauma and stability considerations	[85, 86]
GEFIs	Nanomolar sensitivity; Ratiometric normalization via fusion with constitutive RFP to correct expression-level fluctuations	Real-time dynamic imaging; Genetic targeting to specific cell types and subcellular organelles; Suitable for chronic process monitoring; Limited by tissue autofluorescence	High specificity for H_2_O_2_via OxyR-based sensing domain; C121S mutant as H_2_O_2_-insensitive control to confirm signal specificity	Live cells, primary neurons, brain slices, and living animals; AAV-mediated delivery for stable expression in specific brain regions; Long-term monitoring in transgenic models	[87, 88]

Abbreviations: EPR, Electron Paramagnetic Resonance; DCFH-DA, 2',7'-Dichlorodihydrofluorescein Diacetate; DHE, Dihydroethidium; MitoSOX, Mitochondrial Superoxide Indicator; RFP, Red Fluorescent Protein; AAV, Adeno-Associated Virus; GEFIs, Genetically Encoded Fluorescent Indicators.

In conclusion, excessive OS disrupts SC function via ferroptosis and signaling impairments. However, SCs counteract this redox imbalance through intrinsic antioxidant defenses, a self-rescue mechanism that forms the biological basis for the biomaterial designs discussed in the next section.

## Activation of intracellular antioxidant pathways

### Activation and regulation of the autophagy pathway

#### Molecular mechanisms of the mitophagy pathway

Following recognition by autophagy receptors, damaged mitochondria are sequestered into autophagosomes characterized by a double-membrane structure. These autophagosomes then fuse with lysosomes, where lysosomal hydrolases ultimately degrade the enclosed mitochondria [89]. This process helps alleviate oxidative damage by clearing dysfunctional organelles and degrading protein aggregates, thus limiting further ROS production. It is involved in preserving cellular homeostasis while actively facilitating tissue repair processes within the injury microenvironment [90].

The specific mechanisms are as follows: (1) PTEN-induced putative kinase 1/Parkin (PINK1/Parkin)pathway: OS-induced mitochondrial dysfunction or structural damage reduces ΔΨ_m_, thereby preventing PINK1 translocation into the inner mitochondrial membrane for degradation. Instead, PINK1 accumulates on the outer mitochondrial membrane, where it recruits and activates the E3 ubiquitin ligase Parkin. Parkin, in turn, tags damaged mitochondria by ubiquitinating their outer membrane proteins. Autophagy receptors, such as p62/SQSTM1, subsequently recognize these ubiquitinated proteins and interact with autophagy-related proteins, like LC3, to initiate autophagosome formation. This process ultimately delivers the sequestered mitochondria to lysosomes for degradation [91]. (2) NIX/BCL2 Interacting Protein 3 (BNIP3) pathway: Under OS, the expression levels of BNIP3 and NIX are markedly upregulated. These proteins directly interact with LC3 via their LC3-interacting region (LIR), thereby initiating mitophagy [92]. (3) FUN14 Domain-Containing Protein 1(FUNDC1) pathway: FUNDC1 is an outer mitochondrial membrane protein that primarily initiates mitophagy by interacting with LC3 [93]. These pathways act synergistically to preserve mitochondrial functional homeostasis by eliminating damaged mitochondria and reducing ROS accumulation, thereby playing a pivotal role in maintaining cellular equilibrium and promoting repair following PNI.

#### Bidirectional regulation of macroautophagy in SCs

The AMPK and Mechanistic Target of Rapamycin (mTOR) signaling pathways play key roles in regulating macroautophagy in SCs. The occurrence of OS is accompanied by enhanced SC activity and increased energy consumption. The resulting low-glucose environment enables AMPK to directly activate Unc-51-Like Kinase 1(ULK1) through phosphorylation at Serine 317 and Serine 777. As the key initiator kinase of autophagy, ULK1 promotes autophagy in SCs upon activation to counteract energy stress. Concurrently, ROS elevates the AMP/ATP ratio, which activates AMPK and induces autophagosome formation. This process clears damaged cellular components, thereby reducing further ROS production and exerting an indirect antioxidant effect. However, under nutrient-sufficient conditions (a high-glucose environment), mTOR signaling phosphorylates ULK1 at Ser757 and disrupts the interaction between ULK1 and AMPK, blocking ULK1 activation and preventing excessive autophagy in SCs [94, 95]. Taken together, this mechanism is bidirectionally and precisely regulated by nutrient availability, leading to the activation or inhibition of ULK1 through the AMPK and mTOR signaling pathways, respectively. Although ULK1 regulation produces divergent effects on SCs, the net outcome is the same: maintaining cellular survival and function. Elucidating the interplay between autophagy and antioxidant mechanisms in SCs will aid the development and validation of targeted therapies.

### Synergistic action of enzymatic and non-enzymatic antioxidant systems

SCs contribute to improving the microenvironment at the injury site by eliminating OS products through the activation of both enzymatic and non-enzymatic antioxidant defense systems. When the body is subjected to OS, cells respond by activating the antioxidant enzyme system to upregulate the activity of key antioxidant enzymes [96]. These enzymes neutralize ROS by converting them into non-toxic metabolites, thereby attenuating ROS-induced cellular damage. The key enzymes involved in this defense mechanism include SOD, catalase (CAT), and glutathione peroxidase (GSH-Px) [97]. In parallel, non-enzymatic antioxidants such as vitamin C, vitamin E, vitamin A, melatonin, and GSH directly neutralize ROS to protect neural cells.

The two systems cooperate to regulate the OS environment through enzymatic and non-enzymatic reactions efficiently [98]. Superoxide dismutase (SOD) can convert O_2_^−^ to H_2_O_2_. The low and high levels of H_2_O_2_ scavenging enzymes are Peroxiredoxin (Prx) and CAT, respectively. Glutathione peroxidase (GSH-Px), which utilizes glutathione (GSH) as the electron donor, exhibits a higher affinity for organic peroxides. In addition, antioxidant enzymes such as Glutaredoxin (GRX) and Thioredoxin (Trx), along with non-enzymatic GSH, provide NADPH-driven reducing equivalents. These reducing agents restore oxidized proteins or regenerate antioxidant enzymes through distinct pathways. Through this mechanism, antioxidant enzymes are regenerated and reused, thereby establishing a sustainable cyclic process.

Different from the above macromolecules, vitamin C (Vit C), vitamin E (Vit E), and vitamin A (Vit A) can directly scavenge ROS, which is one of the keys to the antioxidant function of SCs [98–100]. This multi-layered, interwoven defense system strengthens the antioxidant capacity of anti-SCS and enables them to better cope with the OS pathological environment.

### New strategies: regulation of signaling pathways and precise delivery of antioxidants

Excessive ROS *in vivo* can directly or indirectly activate multiple intracellular signaling pathways, influencing cell fate by regulating gene expression. Both pro-survival and pro-apoptotic pathways may be triggered in response to elevated ROS levels [57]. In pro-survival signaling, ROS promote cell survival by the activation of relevant pathways such as Nrf2/ARE, MAPK/Extracellular Signal-Regulatied Kinase (ERK), PI3K/Akt, and NF-κB [101–104]. These pathways upregulate the expression of molecular chaperones. Alternatively, in pro-apoptotic signaling, ROS activate the Janus Kinase (JAK)/Signal Transducer and Activator of Transcription (STAT), Ataxia Telangiectasia (ATM)/p53, and MAPK/c-Jun N-terminal Kinase (JNK)/p38 pathways [46]. This activation enhances the expression of pro-apoptotic proteins such as Bax and Caspase-3, ultimately leading to cell apoptosis. Precise regulation of key signaling nodes is essential to maintain a dynamic balance between pro-survival and pro-apoptotic pathways. This balance represents a central therapeutic target for preserving SC survival. Moreover, this concept underpins the design of antioxidant bioactive scaffolds that mitigate OS-induced damage and promote nerve regeneration.

Related studies provide insights into antioxidant strategies targeting specific signaling pathways. For instance, Zheng et al. demonstrated that MB-CDs activate Nrf2, which subsequently upregulates HO-1 and GPX4 expression. Nrf2 also converts hemoglobin into CO, Fe^2+^, and biliverdin. Biliverdin is then reduced to bilirubin, which exerts antioxidant effects. HO-1 and GPX4 inhibit ferroptosis in SCs by reducing the production of lipid peroxides [30]. Song et al. developed a porous Se@SiO_2_ nanocomposite that sustains selenium release. This material effectively activates the PI3K/AKT pathway, thereby reducing ROS production, suppressing apoptosis, and promoting cell migration and nerve regeneration [75] (Table 2).

**TABLE 2:** Mechanisms of action of relevant signaling pathways.

Categories	Pathways/molecules	Mechanism of activation/intervention	Effector molecules/expression changes	Functions/Results	Related research/applications
Pro-survival pathways	Nrf2/ARE	Activated by oxidants	Increasing expression of NQO1, HO-1, GST (antioxidant enzymes)	Promoting cell survival	[101]
	MAPK/ERK	Activated by growth factors	Upregulating p-MEK, BDNF, and GDNF mRNA expression	Inhibiting apoptosis and promoting neurotrophic factors	[102]
	PI3-K/AKT	Activated by insulin	Reducing thioredoxin-interacting protein (TXNIP) levels	Enhancing SC survival under high-glucose conditions	[104]
​	NF-κB	Activated by TNF-α	Phosphorylating IKKα/β, IκBα, and p65	Facilitating SC survival and nerve regeneration	[103]
Pro-apoptotic pathway	JAK/STAT	Activated by cytokine receptors	Promoting Bax, Caspase-3 (pro-apoptotic proteins)	Inducing apoptosis	[46]
	ATM/p53	Activated by DNA damage	Activating p53 and promoting Bax expression	Triggering an apoptotic program	[46]
	MAPK/JNK/p38	Activated by stress signaling	Activating pro-apoptotic proteins	Promoting apoptosis under OS	[46]
Antioxidant treatment cases	MB-CDs	Activated the Nrf2/HO-1/GPX4 pathway	Up-regulated HO-1 expression and restored GPX4 activity	Enhanced ROS scavenging and improved cell viability Reduced iron-dependent lipid peroxidation	​
	Se@SiO_2_ nanoparticles	Activated the PI3-K/AKT pathway	Upregulating Bcl-2, Nrf2, HO-1 and SOD2; inhibiting Bax	Inhibiting OS and facilitating nerve regeneration	[75]
Collaborative therapeutic strategy	Multi-target pathway activation	Joint activation of Nrf2-Keap, FOXO1 and other pathways	Dynamic balance of pro-survival/pro-apoptotic signaling	Enhances endogenous antioxidant defence	Smart neural repair material design
	Drug delivery systems	Nanotechnology, hydrogel loading technology	Improving antioxidant bioavailability and targeting	Precisely regulates ROS levels to promote nerve regeneration	Slow-release drug scaffolds, spatiotemporal specific modulation systems
	Systemic regulatory framework	Integrating OS signaling networks with multi-target therapy	Synergistic antioxidant-antiapoptotic effects	Realizes multidimensional therapeutic system from molecular mechanism to engineering	Theoretical basis for the development of next-generation intelligent nerve repair materials

Abbreviations: Nrf2, Nuclear Factor Erythroid 2-Related Factor 2; ARE, Antioxidant Response Element; MAPK, Mitogen-Activated Protein Kinase; ERK, Extracellular Signal-Regulated Kinase; PI3-K, Phosphoinositide 3-Kinase; AKT, Protein Kinase B; NF-κB, Nuclear Factor Kappa-Light-Chain-Enhancer of Activated B Cells; JAK, Janus Kinase; STAT, Signal Transducer and Activator of Transcription; ATM, Ataxia Telangiectasia Mutated; p53, Tumor Protein p53; JNK, c-Jun N-Terminal Kinase; p38, p38 Mitogen-Activated Protein Kinase; MB-CDs, Mung Bean-Derived Carbon Dots; Se@SiO_2_, Selenium-Loaded Silica Nanoparticles; GPX4, Glutathione Peroxidase 4; HO-1, Heme Oxygenase-1; SOD2, Superoxide Dismutase 2; FOXO1, Forkhead Box O1.

Notably, the strength of evidence across these pathways is not uniform. The Nrf2/ARE and PI3K/Akt axes are the most consistently supported by *in vivo* data across multiple PNI and DPN models [29, 30, 75, 105], whereas the pro-apoptotic roles of JAK/STAT and ATM/p53 are predominantly context-dependent, with activation reported primarily under specific pathological conditions rather than as a universal response to mechanical nerve injury [46].

The mechanical and topographical cues presented by biomimetic matrices, as cross-referenced in our discussion on bionic ECM scaffolds, are not merely structural; they provide the primary physical stimuli that prime downstream signaling pathways, suggesting that material engineering can be strategically tailored to optimize therapeutic intracellular responses. In summary, SCs combat OS via coordinated autophagy alongside enzymatic and non-enzymatic defenses. Because severe PNI often overwhelms these endogenous pathways, the next section will explore how advanced neural tissue engineering provides targeted exogenous support to amplify these exact mechanisms.

## A new paradigm of neural tissue engineering based on the interaction of oxidative stress in SCs

Peripheral nerve regeneration is a highly dynamic and multifactorial regulated pathophysiologic process [106, 107]. OS, as a key pathological aspect of nerve injury, can affect neuronal survival and axonal regeneration through mechanisms such as regulating intracellular ROS content and interfering with signaling pathways and enzyme activity [25]. In the field of neural tissue engineering, overcoming the limitations of the functional singularity of traditional neural scaffolds remains a major objective. Current research focuses on the development of 3D biomimetic NGCs to build composite regenerative systems with microenvironmental responsiveness by integrating functional cells (e.g., SCs, MSCs, etc.) with smart biomaterials [108].

These novel NGCs not only replicate the spatial architecture of the neural basement membrane but also facilitate axonal regeneration through the controlled release of neurotrophic factors (NGF, BDNF, etc.) alongside antioxidant molecules [109, 110]. The core mechanism lies in the construction of an SC-mediated antioxidant-promoting regeneration molecular system, thus blocking the inhibitory effect of OS on axon growth. Evidence suggests that NGCs enable to remodeling the microenvironment to support axon extension. This is achieved by promoting macrophage polarization toward a reparative phenotype to attenuate the inflammatory response [91, 111].

With the innovation of biomanufacturing technology, the focus of current research on neural tissue engineering has shifted from pure structural biomimicry to a new stage of functionalized synergistic regeneration [112]. Looking ahead, smart NGCs are expected to integrate OS sensing modules with dynamic drug delivery systems to overcome the limitations of the static function of traditional scaffolds. By enabling real-time monitoring of ROS levels in the microenvironment and adaptive regulation of antioxidant release, these advanced systems will achieve more precise and responsive therapeutic interventions. This targeted functional reconstruction strategy facilitates both vascular regeneration and the restoration of bioelectrical signaling. Simultaneously, it allows for accurate control of local redox balance. Collectively, these effects offer a novel solution that bridges anatomical repair with functional recovery in peripheral nerve regeneration. Ongoing advances in this field suggest that neural tissue engineering is shifting from passive structural support toward active microenvironmental regulation, with the ultimate goal of achieving functional regeneration in complex nerve injuries [113–115].

### The design and function of NGCs

#### Optimization of aperture and structure of NGCs

Based on pore size, NGCs can be categorized into large-pore, small-pore, and multi-channel scaffold types. These structural features directly influence cellular behavior, mass exchange, and ultimately the efficacy of nerve regeneration [116–118]. Therefore, designing the ideal pore size of NGCs requires in-depth consideration of its decisive role in determining the fate of Schwann cells within the OS microenvironment. Studies have shown that sufficiently large channels and highly interconnected porous structures can effectively promote the ingrowth of new blood vessels and the exchange of nutrients. [119, 120]. This is critical for reversing the energy depletion of Schwann cells caused by mitochondrial dysfunction under oxidative stress. Furthermore, the open three-dimensional structure provides space for early infiltration of immune cells, particularly macrophages, facilitating their polarization toward the alternatively activated macrophage(M2) pro-regenerative phenotype. This, in turn, accelerates the clearance of myelin debris exacerbated by oxidative stress and fundamentally breaks the vicious cycle of inflammation and oxidative stress [119].

In contrast, scaffolds with excessively small pores, while physically blocking fibroblast invasion and reducing fibrotic scar formation, may simultaneously act as a physical barrier, limiting Schwann cells from obtaining sufficient oxygen and nutrients [119].Under oxidative stress, this nutrient-restricted environment renders Schwann cells more susceptible to ferroptosis, ultimately leading to regeneration failure [121].Thus, an ideal NGC should not be a single homogeneous structure, but rather should possess graded pore sizes or composite pore architectures, so as to ensure the metabolic demands and viability of Schwann cells under stressful conditions while providing topographical guidance.

Based on the above biological requirements, researchers have developed various multilevel channel structures. For example, Maeng et al. constructed a GelMA/PECDA multichannel nerve conduit with good biocompatibility. The 200-μm main channel, combined with micro/nanoporous structures on the inner wall, acted synergistically to significantly enhance nerve regeneration efficiency in animal experiments [122]. Another approach exerts its effects by regulating the cell growth environment. Wang et al. utilized radial and longitudinal foaming techniques to flexibly adjust the pore size distribution of PCL/PVP fiber scaffolds, such that the dimensional variations of the nanoscale grooves effectively guided the directional migration of Schwann cells from the periphery to the center and from the bottom to the top of the scaffold, significantly improving cell infiltration efficiency [123].

In summary, while multichannel conduits excel at providing macroscopic guidance to enhance nerve regeneration efficiency, gradient foaming scaffolds utilize nanoscale groove variations to precisely direct the migration and infiltration of SCs from the periphery to the center. Both strategies demonstrate that over-reliance on uniform pore sizes is problematic, as excessively large channels may lack topographical precision, whereas excessively small pores act as a physical barrier that induces nutrient restriction and SC ferroptosis under oxidative stress. Therefore, the ideal NGC must adopt graded or composite pore architectures to balance vascular nutrient exchange with topographical guidance.

#### Material selection for NGCs

The nature of the material is one of the core elements determining the function of NGCs and is a key research direction in neural tissue engineering [124, 125]. The material system has evolved from natural to synthetic and composite materials, with progressive optimization of their respective performance merits enabling structurally and functionally synergistic integration. Natural materials have garnered significant attention due to their excellent biocompatibility. These materials can be broadly categorized into two types. One type consists of biological tissue-derived materials such asECM. The other type includes natural polymers such as chitosan (CS), collagen, hyaluronic acid (HA), and fibrin [126, 127]. A representative example is CS, whose reactive amino groups along the molecular chain enable diverse chemical modifications, endowing it with excellent biological properties. As a result, CS is considered a highly promising biomaterial for nerve repair. Other natural polymers such as collagen, alginate, and glycosaminoglycans have also shown great potential in this field [128–130]. Li et al. [131] fabricated a nerve conduit by combining biocompatible CS with biodegradable HA. This conduit effectively promoted the proliferation of SCs while inhibiting excessive fibroblast growth, thereby reducing scar formation and tissue adhesion, and ultimately facilitating peripheral nerve repair and regeneration. By comparison, synthetic materials such as polylactic acid (PLA), polyglycolic acid (PGA), and polycaprolactone (PCL) exhibit superior mechanical strength and elasticity [132, 133]. To overcome the limitations of single-material properties, composite materials are designed to integrate the advantages of multiple components, aiming to construct NGCs with enhanced functional performance and improved adaptability to the native tissue environment. Beyond their intrinsic physicochemical properties, the selection of these materials must consider their ability to modulate SC redox homeostasis—a criterion that increasingly guides the development of next-generation NGCs.

In light of the increasingly diverse range of available biomaterials, researchers must establish a comprehensive evaluation framework that explicitly links each material property to SC fate under oxidative stress: (1) Biohistocompatibility: Traditionally, the concept of biohistocompatibility referred primarily to the absence of harmful responses within the host.

Contemporary definitions, however, require more than just compliance with basic safety parameters such as non-toxicity and non-carcinogenicity. Increasingly, emphasis is being placed on the material’s ability to engage in dynamic, bidirectional interactions with host tissues and to adapt to the local biological environment [134, 135]. Jin’s team [136] embedded a urolithin A (UA) hydrogel, which is known for its anti-inflammatory and antioxidant properties, into the lumen of a PCL-based UA NGC. This strategy effectively reduced ROS production and inflammation by leveraging the material’s adaptive response to the microenvironment. (2) Biodegradation rate: The biodegradation rate plays a critical role in determining the pace and effectiveness of nerve regeneration. Fast-degrading materials such as CS, gelatin, and PLGA typically degrade within weeks to months, making them well-suited for acute nerve repair. While slow-degrading materials like PCL may take several months or even years to degrade, they offer prolonged and stable microenvironmental support for the regeneration of long-segmental nerve defects [137–140]. (3) Mechanical properties: Mechanical properties, as fundamental attributes of material performance, play a critical role in ensuring the functional integrity of nerve conduits under complex physiological conditions [141]. Insufficient compressive strength compromises the material’s ability to withstand dynamic pressure and tensile forces during peripheral nerve regeneration, whereas excessive rigidity may inflict mechanical damage on nerve stumps and regenerating microvessels [142]. Type I collagen, for instance, is widely employed as a base material for NGCs due to its excellent biocompatibility, biodegradability, and low immunogenicity. However, its inherently poor mechanical strength poses a risk of structural collapse [143, 144]. To overcome this limitation, Duan et al. [145] developed a mineralized collagen composite conduit by integrating mineralized collagen with type I collagen. This strategy significantly improves the compressive strength of the conduit while maintaining its bioactivity, thereby enhancing its potential for clinical application [146]. This multidimensional evaluation framework calls for researchers to move beyond the limitations of single-performance metrics and achieve synergistic improvements in bio-responsiveness and functional durability through strategies such as biomimetic design and composite modification (Table 3). Each material category inherently presents a distinct trade-off between bioactivity and physical robustness, prompting the field to increasingly favor composite and biomimetic designs that reconcile these competing demands while introducing new challenges in fabrication reproducibility and long-term *in vivo* stability.

**TABLE 3:** Key properties and research progress of various types of nerve guidance catheter materials.

Categorical dimensions	Evaluation index	Key elements	Representative materials/technologies	Research cases/applications
Material type	Natural materials	Tissue-derived materials: ECM, etc.	CS	CS + HA composite catheter → promotes Schwann cell production, inhibits fibroblast proliferation, and reduces scar formation [131]
		Natural polymers: CS, collagen, HA, fibrin		
	Synthetic material	PCL,PLGA	PLGA/PCL	[132]
Rating system	Biological histocompatibility	Uniform standards: Non-toxic, non-carcinogenic	UA hydrogel	UA hydrogel embedded in the lumen of PCL catheter → reduction of ROS and inflammation [136]
		Modern criteria: Bidirectional interaction and adaptability of materials to host tissues		
	Biodegradation rate	Highly degradable material (weeks - months): Suitable for acute nerve repair	CS/gelatine /PLGA	PLGA: Acute repair; PCL: Providing a stable microenvironment for large segmental defects [137–140]
		Low degradation materials (weeks - years): Supporting long-term repair of large nerve defects	PCL	
	Mechanical properties	Insufficient compressive strength: Structure prone to collapse	Type I collagen (natural defect: Insufficient mechanical strength)	Mineralized collagen + type I collagen → increase stress resistance [145]; Bi-layer knitting technology → optimization of mechanical properties and modulation of SCs [146]
		Excessive rigidity: causes mechanical damage		
		Ideal: Balancing dynamic stress with injury avoidance		
Improvement of technology	Composite modified	Skill enhancement through composite/structural design synergy	Mineralized collagen composite catheter, CS/HA composite catheter	[131, 145]
	Bionic design	Modeling the structure and function of the natural neural microenvironment	Double braided conduit	Bilayer structure → maintains stability + promotes controlled release/proliferation/spatial distribution of SCs [146]
Key challenges and responses	Optimization of mechanical properties	Insufficient mechanical strength of natural materials (e.g., collagen) → improvement by composite mineralized collagen or structural design	Mineralized collagen complex catheter	[145]
	Functional synergy enhancement	Biological responsiveness (e.g., anti-inflammatory, antioxidant)	Degradation rate adaptive material selection	UA hydrogel/PCL composite [136]
		Functional durability (degradation-matched regeneration cycle)		

Abbreviations: CS, Chitosan; HA, Hyaluronic Acid; PCL, Polycaprolactone; PLGA, Poly(Lactic-co-Glycolic Acid); UA, Urolithin A; ECM, Extracellular Matrix.

Excessive rigidity disrupts the delicate mechanical microenvironment, which renders SCs increasingly vulnerable to OS [147]. Emerging evidence suggests that the ideal matrix stiffness should mimic the native neural ECM, modulating cell behavior through integrin-mediated mechanotransduction [148, 149]. Furthermore, material mechanical properties function as active regulators rather than passive supports [150]. SCs transduce physical stimuli, such as matrix stiffness or mechanical stretching, into biochemical signals through integrin-mediated mechanotransduction and mechanosensitive channels [141, 150]. This mechanochemical coupling facilitates the homeostasis of pro-survival signaling pathways and metabolic fitness, creating a synergistic framework where material engineering actively participates in biological regeneration.

### Innovative functional neural stents

#### Scaffolds with biologically conductive properties

Electrophysiologic homeostasis of nerve cells is the basis for maintaining the physiologic function of the nervous system [13]. Neuroinjury-induced oxidative stress not only exacerbates microenvironmental deterioration by triggering a surge of proinflammatory cytokines and aberrant protease secretion but also critically disrupts the functional integrity of voltage-gated ion channels, such as Na^+^/K^+^-ATPase [151–153]. Specifically, ROS can induce thiol-oxidizing modifications that alter the conformation of ion or protein channels on the neuronal membrane, resulting in a marked prolongation of the action potential refractory period and consequently disrupting neuronal electrical signaling [154, 155]. This dual disruption of the electrochemical microenvironment initiates a vicious cycle: oxidative stress aggravates environmental deterioration and impairs the myelin regenerative capacity of SCs, and impaired action potential conduction suppresses the secretion of neurotrophic factors. Together, these effects culminate in the failure of axonal regeneration.

Based on this, the design of the new generation of conductive bioscaffolds has evolved from the traditional concept of electrical conductivity to the “electroactivity-modulated” smart materials [156, 157]. Fundamentally, the material must integrate efficient charge transport across the cell-substrate interface with the capacity to modulate both cell-substrate and cell-cell interactions [158–162]. These materials contribute to the optimization of the nerve regeneration microenvironment through several mechanisms. They promote the adhesion of SCs and growth factors, facilitating initial cellular integration. They also modulate cell–matrix interactions to fine-tune the scaffold’s molecular architecture and surface charge distribution. Moreover, they enable functional electrical coupling between the scaffold and host tissues, which is essential for neuromodulation and signal conduction [163]. For example, graphene-based composite scaffolds, owing to their unique sp^2^-hybridized orbital structure, can establish a three-dimensional conductive network that promotes electrical coupling among SCs. Additionally, the abundant π-electron cloud on their surface enables efficient scavenging of oxidative species such as OH^−^, thereby achieving a synergistic enhancement of both electrical conductivity and antioxidant capacity [162, 164, 165].

Biologically, this graphene-mediated microenvironment optimization facilitates the dual activation of Schwann cells in the peripheral nervous system and astroglia in the central nervous system. *In vivo* evidence confirms that this translates to robust remyelination by upregulated MBP and restored functional electrical coupling, significantly increasing nerve conducting velocity and compound motor action potentials compared to pure PCL controls [165].

Moreover, conductive scaffolds are not merely physical conductors of electrical current; more importantly, the weak electrical signals they induce can act as exogenous biophysical signals that activate endogenous calcium signalling in Schwann cells [166] and downstream FAK/AKT pro-survival pathways [167]. This, in turn, counteracts the OS microenvironment [74] and ultimately reverses the OS-induced suppression of neurotrophic factor (e.g., BDNF and NGF) secretion by Schwann cells [166, 167]. Notably, graphene-based scaffolds distinguish themselves from most conductive polymers by offering concurrent charge transfer and intrinsic ROS scavenging, whereas other conductive materials predominantly rely on exogenous biophysical stimulation to indirectly alleviate oxidative stress. This distinction underscores the growing importance of multifunctionality in scaffold design for the OS-compromised microenvironment.

#### Biomimetic ECM scaffolds

Compared with conventional scaffolds, biomimetic ECM scaffolds mimic physiological structures, not only restoring anatomical integrity but also enabling targeted functional reconstruction [168]. Bionic ECM scaffolds reconstruct nanofiber networks mimicking basement membrane pore size and mechanical gradients via electrospinning, 3D printing, and other techniques, thereby accelerating guided axon regeneration and reducing ROS exposure time [169–171]. Such scaffolds provide not only physical support for regenerating tissues but also act as dynamic signaling platforms. Through the multimodal integration of bioactive molecules, anti-inflammatory agents, antioxidants, and conductive components, they enable precise regulation of the regenerative microenvironment [127, 172, 173].

As discussed in earlier sections, the OS environment induces mitochondrial metabolic dysfunction and homeostatic imbalance in SCs. Jiang et al. fabricated a multilayered conduit combining PCL nanofiber topology with blend-loaded melatonin (MLT) and reduced graphene oxide (RGO). Biologically, while RGO restores bioelectrical conduction, MLT maintains a stable ΔΨ_m_ as confirmed by JC-1 assay, which bioenergetically prevents the proton motive force reversal to abort the RET-induced ROS burst at Complex I and III. This metabolic rescue amplifies ATP supply for energy-demanding myelin regeneration, achieving functional and morphological recovery close to autologous nerve grafts in a 10-mm rat sciatic nerve defect model. Beyond chemical ROS-scavenging designs, the anisotropic physical topology of biomimetic ECMs (e.g., aligned nanofibers and microgrooves) independently drives directional SC migration and upregulates crucial myelination-associated genes, including *Mbp* and *Sox10*. Mechanistically, this topographic guidance running via cytoskeleton-mediated mechanotransduction specifically targets and activates three major intracellular cascades: the Wnt/β-catenin, ERK/MAPK, and TGF-β signaling pathways [174]. This biophysical regulation runs parallel and complementary to biochemical antioxidant signaling, cooperatively rescuing SC functions and accelerating re-myelination within the hostile oxidative niche.

The bionic ECM scaffold, with its multidimensional bionic advantages in structure, signal, and function, is promoting the upgrade of peripheral nerve repair from “passive support” to “intelligent guidance.” Chen et al.'s composite conduit actively scavenges ROS via disulfide bonds. This suppresses NF-κB/IL-1β and restores PI3K/AKT/mTOR signaling, which promotes Schwann cell proliferation and angiogenesis. Consequently, the repair strategy shifts from passive guidance to active microenvironmental reprogramming. [175]. Through the biomimicry of the native microenvironment and the integration of multimodal regulatory strategies, this approach improves both the spatial and temporal precision of axonal regeneration. It thereby establishes a robust foundation for the construction of personalized nerve grafts capable of achieving tissue integration and functional restoration [176].

These biomimetic ECM strategies promote regeneration through distinct yet complementary mechanisms: physical anisotropic topologies directly drive SC migration and myelination gene expression via mechanotransduction signaling, whereas biochemical modifications actively scavenge ROS, prevent RET-induced oxidative bursts, and restore bioelectrical conduction. Although combining these physical and biochemical cues successfully shifts nerve repair from passive guidance to active microenvironmental reprogramming, realizing a clinically feasible solution still requires further optimization of the dynamic coupling between material degradation and factor release, as well as maintaining the long-term stability of the scaffold-cell interface.

### The new concept of intelligent drug delivery system

**FIGURE 6:**
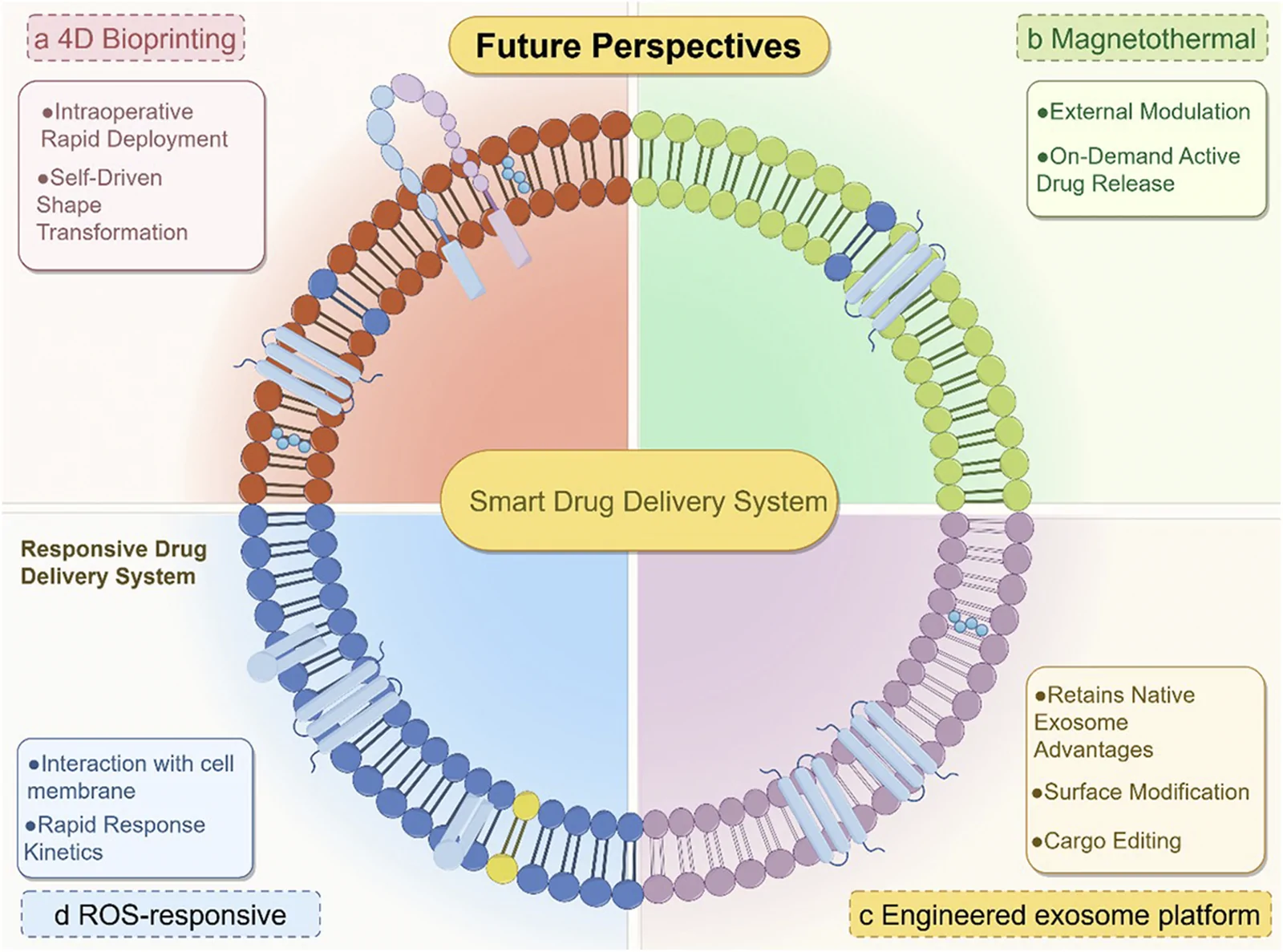
**(a)** The ROS-responsive drug-controlled release system offers high targeting specificity and enables rapid response to injury-induced ROS in early stages; **(b)** the magnetothermal controlled-release system permits external remote activation for on-demand drug release; **(c)** the engineered exosome platform retains the innate targeting capability, biocompatibility, and complex biomolecule-delivery functions of natural exosomes while allowing programmable surface modifications and cargo editing; and **(d)** 4D bioprinting facilitates rapid in vivo formation of NGCs with self-morphing capabilities via hydration-driven expansion or programmed design. Collectively, these approaches promote peripheral nerve regeneration through distinct mechanisms.

A drug delivery system (DDS) refers to the use of specific technical means to precisely control the timing, location, and dosage of drug release, delivering drugs to target tissues or organs in a controlled, efficient, and safe manner to enhance drug efficacy and reduce side effects. Its core advantages lie in improving drug stability, optimizing distribution characteristics, and reducing side effects [135, 136]. Conventional drug delivery systems lack precise spatiotemporal control, which hinders their ability to achieve specific targeting and optimal biodistribution of therapeutic agents [177, 178]. In contrast, intelligent drug delivery systems are capable of dynamically recognizing the lesion microenvironment, performing subcellular targeting, and achieving programmed release in response to endogenous signals (e.g., pH, enzymes, temperature) or external stimuli (e.g., light, ultrasound, magnetic fields). These systems complete a closed-loop delivery process, from protecting drug activity to enabling precise spatiotemporal regulation [3, 113, 179]. In response to the OS microenvironment, the development of ROS-responsive drug-controlled release systems, magnetothermal-regulated release systems, and engineered exosome-based delivery platforms holds significant potential for mitigating ROS-induced damage and enhancing the proliferation of SCs (as shown in Figure 6).

#### ROS-responsive drug-controlled-release system

The significant increase in ROS levels at the site of nerve injury provides a theoretical basis for the construction of a ROS-responsive drug-controlled release system. Such systems are typically engineered with ROS-sensitive linkers to create stable ROS-responsive materials. They may also incorporate hydrogels or nanoparticle-based carriers loaded with functional cells, neurotrophic factors, antioxidants, and anti-inflammatory agents to enable intelligent stimulus-responsive release and precise therapeutic modulation [180, 181]. ROS-responsive drug piggyback systems vary in the drugs that researchers choose to piggyback. According to the different purposes, they can be broadly categorized into the following four types: (1) Antioxidants can effectively scavenge free radicals and reduce ROS levels, thereby reducing oxidative stress and cell damage [182, 183]. (2) NGF plays a critical role in the nervous system. It facilitates the elongation of neuronal axons and also contributes to the formation of myelin sheaths, both of which are essential for nerve repair and signal conduction [8, 171]. (3) Anti-inflammatory drugs reduce the local inflammatory response by down-regulating pro-inflammatory factors such as TNF-α, IL-6, and IL1-β. Moreover, since OS and inflammation are mutually reinforcing, controlling inflammation can in turn alleviate oxidative stress, thereby further breaking the vicious cycle [60, 133, 184]. [4] Gene therapy drugs can act directly on key pathological links after nerve injury, for example, PINK1 mRNA enters the cell and directs the synthesis of PINK1 protein, which is closely related to mitochondrial autophagy [185].

The above strategies facilitate the diverse development of ROS-responsive systems. o precisely modulate the oxidative and senescent microenvironment, Zhao et al. developed a ROS/Ca^2+^-responsive dynamic hydrogel composed of HA-PBA, TPA, and CS-EDTA. [185]. This hydrogel delivers engineered extracellular vesicles (EVs) loaded with PINK1 mRNA. Through dual modifications, these EVs specifically target DPP4 receptors on senescent SCs. Crucially, once internalized, the PINK1 mRNA directly activates the PINK1/Parkin-mediated mitophagy pathway, which clears damaged mitochondria and halts ROS accumulation. This targeted intervention reverses SC senescence and restores pro-regenerative capacity, thereby accelerating nerve repair. In a complementary approach, Dong et al. engineered a ROS-responsive and thermosensitive hydrogel system (PTCM@PMet nanoparticles embedded within mPEG-PA-PP) to selectively deliver the H_2_S donor, peroxyTCM. Triggered by the high-ROS injury microenvironment, peroxyTCM releases H_2_S. Of note, beyond acting as a direct chemical ROS scavenger, H_2_S serves as a gaseous signaling molecule that is known to activate the Nrf2/HO-1 antioxidant axis and suppress NF-κB-mediated inflammation. This dual-pathway modulation is expected to protect Schwann cells from lipid peroxidation-induced injury, decisively reshaping the hostile oxidative niche into a pro-regenerative milieu [115]. Moreover, this experiment employed the fluorescent probe 7-nitro-4-methylcoumarin (AzMC) to monitor and visualize the accumulation of H_2_O_2_ and H_2_S in the cellular environment, thereby providing a more intuitive reflection of the actual ROS response.

Overall, the ROS-responsive controlled release system offers a novel approach to precisely regulate oxidative stress and inflammation following nerve injury through the synergistic integration of material design and drug loading. Nevertheless, it is worth noting that uncertainties remain regarding the specificity of the response and the persistence of efficacy under multiple pathological stimuli. Consequently, related research needs to further focus on assessing stability and practicality under complex *in vivo* environments [186].

#### Engineering exosome delivery platform

Exosomes are nanoscale vesicles originating from endosomal compartments that carry various biologically active molecules, including RNA and proteins. Through non-cell-autonomous mechanisms, they regulate key cellular processes such as migration, differentiation, and responses to tissue injury, thereby promoting neurite outgrowth and axonal regeneration in the peripheral nervous system [187–189]. Recent studies have shown that exosomes derived from SCs can deliver miR-21 to specifically suppress PTEN expression, thereby relieving the inhibition of the PI3K/Akt signaling pathway. This activation subsequently upregulates anti-apoptotic proteins, promoting neuronal survival and axonal regeneration [190].

In recent years, engineering exosomes to enhance their own functions or improve their targeting has gradually become a new development trend. The engineering approaches that can be employed include direct modification of exosomes, induction of cell differentiation, and pretreatment of parent cells, among others [191]. Applying this direct modification strategy, Liu et al. loaded polyphenol-engineered Saccharina japonica exosomes (CA@Exos) onto electroconductive microneedles to achieve deep-tissue delivery [192]. Concurrently, the application of exogenous electrical stimulation via this bioelectric scaffold significantly amplifies the cellular uptake of these exosomes. Mechanistically, the released caffeic acid (CA) specifically inhibits the formation of AGEs and blocks AGE-RAGE interactions, while mitigating ROS accumulation through the upregulation of antioxidant enzymes such as Sod2 and Hmox1. Furthermore, the combined bio-electroceutical intervention facilitates Ca^2+^ influx, activating Camk2a/Camk2b-mediated Erk1/2 and cGMP-PKG signaling cascades. This targeted multi-pathway modulation fosters robust crosstalk between Schwann cells and vascular endothelial cells. Ultimately, this seamless coupling of redox homeostasis with neuroangiogenesis establishes a self-amplifying loop that significantly accelerates chronic tissue repair [192]. These examples underscore the versatility of exosome engineering: by coupling innate targeting with tailored cargo and surface modifications, exosome platforms can be precisely configured to counteract specific oxidative and inflammatory pathologies in the injured nerve microenvironment.

#### Magneto-thermally controlled release system

The magnetothermal controlled-release system, leveraging localized heating of magnetic nanomaterials under an alternating magnetic field, enables precise delivery of neurogenerative factors and coordinated regulation of the OS microenvironment [193–195]. This feature is conducive to loading magnetic nanoparticles, exosomes, neurotrophic factors, or antioxidants together in a thermosensitive hydrogel. By using the magnetic field-triggered thermal energy, the swelling-shrinking behavior of the hydrogel can be dynamically regulated to achieve the on-demand release of active substances [196–198]. Additionally, magnetothermal stimulation activates TRPV1 channels, leading to a calcium ion influx that promotes Schwann cell migration and axonal growth. Beyond triggering drug release, magnetothermal cues directly activate specific intracellular pathways. For instance, magnetic composite scaffolds can activate the Heat Shock Protein 70 pathway to enhance synergistic tissue regeneration [199]. Furthermore, Kuznetsova et al. engineered a magnetic fibrin hydrogel that generates localized heating upon alternating magnetic field (AMF) exposure. This remote thermal cue specifically targets thermosensitive TRPV1 ion channels, triggering a Ca^2+^ influx that promotes 3D neurite outgrowth and axonal extension [200].

Although systematic application of TRPV1-targeted magnetothermal effects to NGC design remains in its early stages, the recent demonstration of magnetic fibrin hydrogels as implantable neural interfaces [200] provides a strong proof-of-concept that this modality can be integrated into next-generation nerve scaffolds, warranting further mechanistic investigation.

#### 4D bioprinting technology

3D bioprinting can be used to fabricate biocompatible and functionally active neural scaffolds and offers innovative solutions to meet complex neural tissue repair needs by precisely modulating the spatial distribution of cells and biomaterials. Leveraging computer-aided design, 3D printing enables the personalized fabrication of structures with physiological shapes and microstructural features, better meeting the needs of individual patients [201, 202]. Despite its considerable potential, current 3D printing technology still faces significant limitations. One key issue lies in its inability to fully replicate and integrate the complex multimodal signals present in the physiological microenvironment of implantable nerve conduits. Moreover, effectively coordinating stem cell differentiation within these constructs remains a major challenge.

4D bioprinting builds upon 3D printing by introducing a time-dependent dynamic response. This technology enables printed structures to adapt to physiological environments in real time. It achieves this adaptability by combining smart-responsive materials such as temperature-sensitive hydrogels, light-responsive hydrogels, and shape memory polymers with preprogrammed structural designs. Examples of such designs include anisotropic shrinkage, gradient degradation, and predesigned deformation patterns. The core breakthroughs are reflected in three key aspects: (1) By studying and mimicking the dynamic changes of key structures such as the extracellular matrix during the nerve regeneration process, researchers can reconstruct a functional microenvironment at the injury site. This reconstructed microenvironment is not fixed in form. Instead, it can continuously adapt and remodel itself in coordination with the distinct stages of nerve regeneration. Such adaptability ensures sustained support for cellular activity and promotes progressive tissue repair. (2) Employ computational programming to precisely regulate the spatiotemporal release of neurotrophic factors and antioxidants. This enables the deliberate setting of parameters such as dosage, timing, and release location, facilitating alignment with the evolving demands of nerve regeneration. (3) Integrate electrically active scaffolds with conventional biological scaffolds that provide structural support and directional guidance, thereby synergistically enhancing the conduction of electrical signals between neurons [203–206].

Currently, the application of 4D bioprinting specifically targeting OS in nerve repair remains largely unexplored. However, recent dynamic designs provide a strong foundational framework. For example, Wang et al. developed a 4D-printed shape-memory conduit (PLATMC/Ti_3_C_2_T_x_ MXene) that autonomously self-rolls at 37 °C within 20 s to rapidly wrap injured nerves [206]. Mechanistically, its pre-programmed microchannels offer topographical guidance for directional SC migration via cytoskeleton-mediated mechanotransduction, while the conductive Ti_3_C_2_T_x_ matrix promotes SC maturation and robust angiogenesis. Although their study focused on biophysical cues, Ti_3_C_2_T_x_ intrinsically possesses potent ROS-scavenging properties, attributed to its inherently reducing Ti-C skeleton and antioxidant enzyme-mimicking properties (e.g., SOD-like and CAT-like activities) [207, 208]. This reveals a promising, untapped strategy: integrating such nanomaterials into 4D-printed scaffolds can synergistically couple morphological self-adaptation with active OS neutralization to comprehensively remodel the hostile regenerative microenvironment.

To systematically summarize these cutting-edge strategies, the representative applications of smart biomaterial systems (including external physical field stimulation, magneto-thermal controlled release, and 4D bioprinting) for ROS scavenging and nerve regeneration are summarized in Table 4.

**TABLE 4:** Summary of antioxidant strategies and structural.

Antioxidant strategy/Mechanism	Related biomaterial/Vehicle	Main function and effect	In-text instance/Source
Activating endogenous antioxidant pathways to inhibit cell ferroptosis	MB-CDs	Activates Nrf2 protein, initiating downstream expression of HO^−1^ and GPX4, reducing the production of lipid peroxides, and inhibiting ferroptosis in SCs	[30]
Sustained release of active elements to activate pro-survival signaling pathways	Porous Se@SiO_2_ nanocomposite	Effectively activates the PI3K/AKT signaling pathway through the sustained release of selenium, thereby reducing ROS generation, suppressing apoptosis, and promoting cell migration and nerve regeneration	[75]
Environment-responsive adaptive regulation to combine anti-inflammatory and antioxidant effects	UA hydrogel embedded into the lumen of a PCL-based NGC	Reduces ROS production and inflammation by leveraging the material’s adaptive response to the microenvironment	[136]
Direct ROS scavenging via surface electron clouds combined with bioelectrical coupling	Graphene-based composite scaffolds/RGO	The abundant π-electron cloud on the surface enables efficient scavenging of oxidative species such as OH^−^; establishes a 3D conductive network to promote electrical coupling among SCs and increase nerve conducting velocity	[162, 165]
Multimodal metabolic rescue (antioxidant release + Bioelectrical conduction)	Multilayered conduit combining PCL nanofiber topology with blend-loaded MLT and RGO	RGO restores bioelectrical conduction, while MLT maintains a stable ΔΨ_m_, preventing the RET-induced ROS burst and amplifying ATP supply for myelin regeneration	​
Active ROS scavenging via chemical bond cleavage to reprogram the microenvironment	Composite conduit actively scavenging ROS via disulfide bonds	Scavenges ROS via disulfide bonds, suppressing NF-κB/IL-1 and restoring PI3K/AKT/mTOR signaling to promote Schwann cell proliferation and angiogenesis	[175]
Dual-responsive stimulus release for subcellular targeted gene therapy	ROS/Ca^2+^-responsive dynamic hydrogel formed by HA-PBA, TPA, and CS-EDTA (delivering engineered EVs loaded with PINK1 mRNA)	Specifically targets DPP4 receptors on senescent SCs; the internalized PINK1 mRNA directly activates the PINK1/Parkin-mediated mitophagy pathway to clear damaged mitochondria, halt ROS accumulation, and reverse SC senescence	[185]
Intelligent controlled-release of gaseous signaling molecules for dual-pathway modulation	ROS-responsive and thermosensitive hydrogel system (PTCM@PMet nanoparticles embedded within mPEG-PA-PP)	Triggered by the high-ROS injury microenvironment to release H_2_S, which acts as a gaseous signaling molecule to activate the Nrf2/HO^−1^ antioxidant axis and suppress NF-κB-mediated inflammation, protecting SCs from lipid peroxidation	​
Engineered exosome delivery platform coupled with external physical stimulation	Polyphenol-engineered Saccharina japonica exosomes (CA@Exos) integrated into electroconductive microneedles	External electrical stimulation amplifies cellular uptake of exosomes, which specifically activate Camk2a/Camk2b-mediated Erk1/2 and cGMP-PKG signaling cascades to efficiently clear ROS and AGEs	[192]
Magneto-thermally controlled release system: Precise delivery of neurogenerative factors and coordinated regulation of the OS microenvironment	Magnetic composite scaffolds/Magnetic fibrin hydrogel (or loading magnetic nanoparticles, exosomes, neurotrophic factors, or antioxidants together in a thermosensitive hydrogel)	Uses magnetic field-triggered thermal energy to dynamically regulate the swelling-shrinking behavior of the hydrogel for on-demand release of active substances. Magnetothermal cues activate the heat Shock Protein 70 pathway and target thermosensitive TRPV1 ion channels, triggering a Ca^2+^ influx that promotes Schwann cell migration, 3D neurite outgrowth, and axonal extension	[199, 200]
4D bioprinting self-adaptation coupled with intrinsic inorganic antioxidant framework	4D-printed shape-memory conduit (PLATMC/Ti3C2T_X_ MXene)	Autonomously self-rolls at 37 °C to rapidly wrap injured nerves; the inorganic matrix (Ti_3_C_2_T_X_) intrinsically possesses potent ROS-scavenging properties due to its reducing Ti-C skeleton and antioxidant enzyme-mimicking properties (SOD-like and CAT-like activities)	[206–208]

Abbreviations: MB-CDs, Mung bean-derived carbon dots; SCs, Schwann cells; UA, Urolithin A; RGO, reduced graphene oxide; MLT, melatonin; AGEs, advanced glycation end products; membrane potential, ΔΨm.

In summary, tissue engineering has evolved from passive conduits to active, intelligent delivery systems capable of precise ROS regulation. These advanced engineering milestones establish a robust foundation for the targeted translational applications discussed next.

## Novel approaches and therapeutic strategies for SCs-OS interactions in PNI/peripheral neuropathy

SCs are key functional units of the peripheral nervous system and play an important role in common peripheral nerve diseases. Whether the peripheral nerves are affected by acute injury or chronic lesions, SCs are involved in the process of nerve repair [209]. Meanwhile, almost all neurological diseases are associated with inflammation, and the cascade effect of OS and inflammation is a common pathological axis running through many peripheral neurological diseases; SCs are not only a key target of this pathological process, but also participate in shaping the center of microenvironmental regulation of the disease through the dynamic interactions between SCs and the immune system [210]. Notably, although the OS-SCs-immune cells “triangle” is prevalent, its specific pattern and pathological outcome are highly dependent on the disease-specific microenvironment [211]. The subsequent discussion will use PNI, DPN, and NF1 as disease models to explore the effects of OS-SCs interactions in peripheral nerve mechanical injuries, metabolic lesions, and hereditary lesions, respectively, and then focus on the precise antioxidant therapeutic strategies developed based on these mechanisms, to provide theoretical bases and prospective perspectives for the future direction of treatment.

While the last section outlined universal tissue engineering strategies, clinical success requires tailoring them to these specific microenvironments. Therefore, before exploring the clinical landscape, we should examine how OS-SC interactions differ across mechanical, metabolic, and hereditary neuropathies, using PNI, DPN, and NF1 as specific models. This will guide the design of targeted precision therapies and provide theoretical bases for future treatments.

### Pathological mechanisms of SCs and oxidative stress in PNI/peripheral neuropathy

#### The interaction mechanism of OS-SCs in PNI

Following mechanical injury to peripheral nerves, SCs are one of the earliest cell types to respond to injury [13]. By inducing mitochondrial dysfunction and DNA damage, oxidative stress not only directly impairs the repair phenotype of SCs, but more crucially, drives their transition from pro-regenerative “repair SCs” to inhibitory “senescent SCs,” thereby deteriorating the regenerative microenvironment [4, 27]. This process is regulated by the Nrf2 antioxidant pathway; insufficient Nrf2 activity accelerates the senescent transition of SCs, whereas Nrf2 activation helps sustain the repair phenotype and resist senescence [30, 105].

Specifically, SCs upregulate the expression of pro-inflammatory factors such as tumor necrosis factor-α (TNFα) and interleukin-6 (IL-6), along with chemokines such as CCL2, through perceived destructive signals in the microenvironment [212, 213]. Subsequently, the above factors recruit monocytes/macrophages to the site of injury, and the polarization of classically activated macrophage (M1) promotes the development of inflammation [214]. At the same time, Nox in SCs, M1, and neutrophils is activated and releases large amounts of ROS. For repair Schwann cells, moderate ROS levels serve as essential second messengers that sustain c-Jun expression and maintain their dedifferentiated state, as evidenced by studies showing that H_2_O_2_ directly modulates c-Jun and ERK activation in injured nerves [69], and that c-Jun is the master transcriptional regulator of the Schwann cell repair program [215]. However, excessive ROS triggers axonal growth cone collapse and retraction, a positive feedback between inflammation and OS formation that is a key component of Wallerian degeneration [9, 38]. The large amount of ROS generated during this process damages the myelin debris produced by SCs, which will impede nerve regeneration. SCs, macrophages, and neutrophils are all capable of phagocytosing and removing myelin debris [216, 217]. As inflammation progresses into the chronic phase, SCs induce polarization of M1 toward M2, which secretes vascular endothelial growth factor (VEGF) with pro-angiogenic effects [210, 214]. Demyelinated SCs form “Büngner bands” along the trajectory of neovascularization, providing structural guidance for axonal regeneration [218, 219].

**FIGURE 7:**
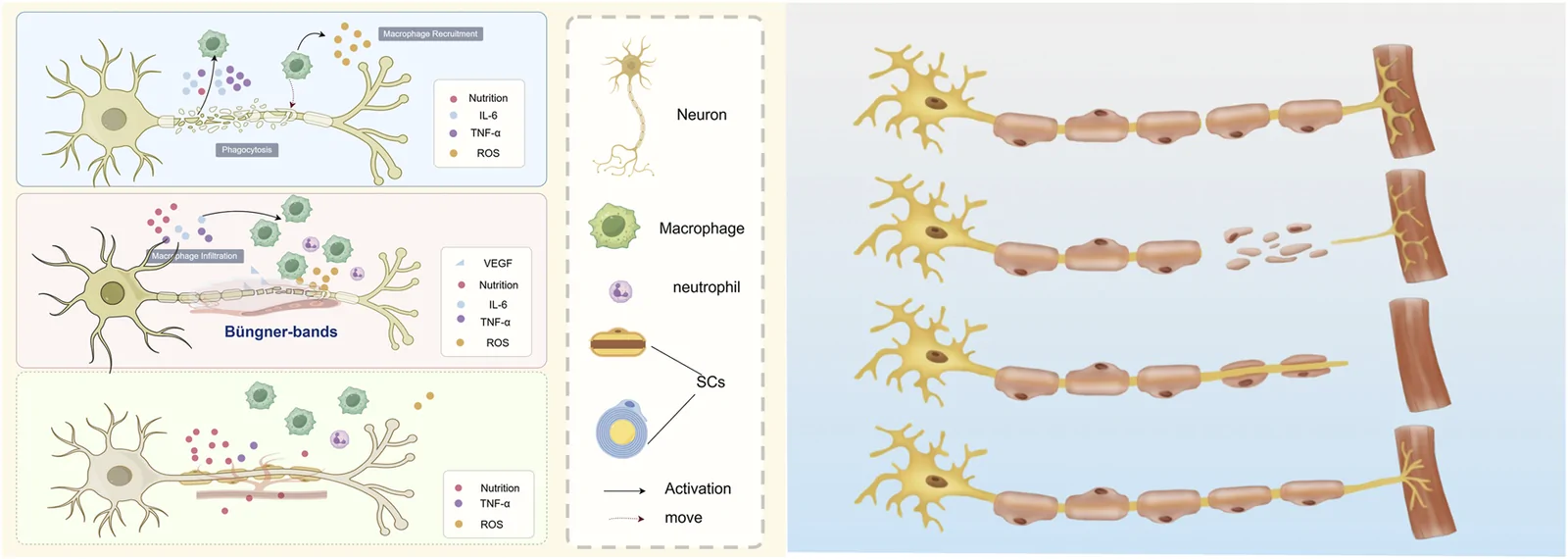
The mechanism of OS and the inflammatory cascade reaction: (1) After PNI, Schwann cells release inflammatory and trophic factors and phagocytose myelin debris. Inflammatory cues recruit macrophages and neutrophil that secrete ROS. (2) Wallerian Degeneration: Axons degenerate, Bungner bands form, and macrophages secrete VEGF to promote angiogenesis. Then SCs migrate along the newly formed vessels. (3) Nerve Regeneration (ideal situation): Axons regrow, ROS and inflammation decrease, and neurotrophic support increases.

Studies have shown that after PNI occurs, the expression of the key antioxidant defense pathway Nrf2 in SCs is downregulated, resulting in a decrease in their antioxidant capacity [64]. This change, to some extent, helps to establish the Wallerian degeneration microenvironment, thereby supporting the process of peripheral nerve repair. In addition, SCs secrete various neurotrophic factors, which regulate the survival, migration, adhesion, and chemotaxis of cells, thereby further promoting axonal regeneration [220] (as shown in Figure 7). The above-mentioned cascade reaction composed of inflammation and OS is not only a classic pathological change after PNI, but also one of the core theoretical foundations for guiding antioxidant treatment.

Although the inflammation-OS cascade is a ubiquitous pathological mechanism following PNI [10], it must be emphasized that different experimental PNI models exhibit divergent characteristics. In the nerve crush model, the basal lamina surrounding the axons remains intact, allowing axons to regrow within their original basal lamina tubes. In contrast, in the cut injury model, the connective tissue and basal lamina tubes are disrupted, compelling regenerating axons to cross the tissue bridge formed between the severed stumps [221]. Therefore, PNI should not be treated as a homogeneous pathological condition. Clearly distinguishing the microenvironmental disparities among these injury models holds significant clinical translational value, suggesting that treatment strategies for peripheral nerve injury must shift toward precision and customized therapy [222].

#### The interaction mechanism of OS-SCs in DPN

DPN is a typical metabolic peripheral neuropathy, and one of the core pathologic bases of its pathologic basis lies in the metabolic disorders and OS triggered by chronic hyperglycemia [223]. Redundant glucose is shifted to the polyol pathway for metabolism due to increased hexokinase activity in the high glucose environment. At this point, SCs metabolize glucose to fructose with the vascular endothelium-expressed aldose reductase AR, which depletes nicotinamide adenine dinucleotide phosphate (NADPH). Decreased levels of NADPH impede GSH synthesis, and cellular antioxidant capacity decreases [66]. Additionally, the Maillard reaction, polyol pathway, ketone body metabolism, and Wolff pathway produce AGEs. Hyperglycemic Glucose (HG) leads to an abnormal accumulation of AGEs [224]. Then, an abnormal accumulation of AGEs results in binding to RAGE on SCs and activating pathways such as NF-κB, JNK, and NLRP3, generating ROS and inflammatory factors that lead to SC damage and dysfunction [225, 226]. Not only that, the HG environment also activates signaling pathways such as PKC [227]. Together, the above pathways promote OS onset and further elicit an inflammatory infiltrate.

A study showed that the infiltration of M1 and resting CD4^+^ memory T cells was increased under DPN conditions. Meanwhile, the infiltration of M2, resting mast cells, monocytes, and T follicular helper cells was decreased, which may be related to the development of DPN [132]. Moreover, *in vitro* studies have shown that high glucose conditions promote sustained M1 polarization of macrophages by downregulating IRG1 expression, suggesting a potential mechanism by which M1 macrophages may persist in diabetic complications such as DPN [228]. However, direct evidence for increased M1 infiltration and decreased M2 in DPN development remains to be established. Also, HG-induced mitochondrial dysfunction and inhibition of the Nrf2 pathway resulted in a decrease in the antioxidant capacity of SCs and a weakening of the ability to withstand stress [229, 230]. As for mast cells, HG-stimulated secretion of factors inhibits myelin formation in SCs [231]. In summary, OS-inflammation co-propels DPN progression. The major cells involved in the process are similar to those of PNI, but the pathologic changes differ in different microenvironments. In general, cells can mitigate greater damage from stressful environments through autophagy. However, HG inhibits the autophagy function of SCs, which is detrimental to the remodeling of the microenvironment [94]. Therefore, modulating the relevant signaling pathways and restoring autophagy in SCs is a promising therapeutic strategy.

#### The interaction mechanism of OS-SCs in NF1

SCs are the origin of NF1, and their tumorigenesis is due to mutations in the NF1 gene within SCs [232]. The mutation results in the deletion of the protein encoding the NF1 gene that inhibits Ras activity, and sustained activation of the Ras signaling pathway. Downstream signaling pathways (e.g., Raf-MEK-ERK, PI3K-Akt-mTOR, etc.) are subsequently activated, promoting the proliferation, migration, and survival of SCs [233]. At the same time, SCs secrete large amounts of Kit ligand to recruit both NF1 mast cells and CSF1 to recruit macrophages. Both NF1 mast cells and macrophages promote the migration and proliferation of SCs by inducing angiogenesis, providing nutrient support, and secreting large amounts of TGFβ to stimulate collagen production by fibroblasts and the formation of tumor strom [234–236]. In conclusion, the pathological microenvironmental regulation of NF1 by SCs-immune cells influences tumor formation to a certain extent, and the study of the mechanisms of cell-cell interactions contributes to the understanding of disease onset and progression. Additionally, activation of signaling pathways such as mTOR has been observed to upregulate ROS production in several studies [16, 237]. Moreover, ROS plays a dual role in cancer. On the one hand, ROS are carcinogenic risk factors in their own right and may also cause damage to the DNA of cells. On the other hand, the cytotoxicity of excess ROS can also act on tumor cells [16]. Therefore, modulation of OS-related signaling pathways in NF1 to intervene in NF1 may serve as a new therapeutic tool.

### Precision antioxidant therapy based on OS-SCs interaction mechanism

In antioxidant treatment for PNI, traditional drugs such as vitamins, curcumin, and alpha-lipoic acid can reduce ROS to some extent [99, 238, 239]. However, these drugs generally lack selectivity for specific cell types and pathways and are considered broad-spectrum, non-targeted therapies. As research on the molecular mechanisms of PNI progresses, antioxidant strategies are evolving toward more precise and targeted approaches.

For instance, Yadav et al. used the Histone Deacetylase (HDAC) inhibitor PBA to verify its repair efficacy in the RT4 SC inflammation model and the sciatic nerve transection model [240]. The results showed that PBA could significantly reduce the expression and secretion of TNF-α and inhibit the phosphorylation of NF-κB-p65. It effectively alleviated the inflammatory response both *in vivo* and *in vitro*. Yao et al. utilized tFNAs as carriers and loaded resveratrol by intercalation to construct a tetrahedral framework nucleic acids (tFNAs)-RSV complex. The complex significantly improved the stability, bioavailability, and cellular uptake efficiency of RSV, and ameliorated the pathological changes and symptoms of DPN by regulating redox balance and mitochondrial bioenergetic metabolism [241]. In contrast to the above strategies aimed at protecting SCs and attenuating oxidative damage, NF1, a tumor originating from SCs, was treated by combining mTOR/HDAC inhibitors with simultaneous inhibition of the two major antioxidant pathways, GSH and TRX, which resulted in dramatic accumulation of ROS and ultimately led to tumor cell death [237].

Furthermore, nerve tissue engineering also provides a new approach for targeted antioxidant therapy of PNI. Li et al. developed a multifunctional delivery system based tFNAs and microRNA-22. It enhances the communication between SCs and macrophages to reconstruct the damaged microenvironment, inhibits inflammation, and significantly reduces ROS expression. At the same time, this system can also upregulate the BDNF/Tropomyosin Receptor Kinase (TrkB) signaling pathway to promote the functional recovery of peripheral nerve injuries [242]. In another study, Wei et al. utilized a light-responsive small spherical algae hydrogel (C-Gel) to regulate local oxygen release and the immune microenvironment. This approach successfully reduced the expression of ROS, TNF-α, IL-6, and Hypoxia-Inducible Factor-1α(HIF-1α), which also promoted the polarization of macrophages from M1 to M2. Consequently, these changes enhanced the proliferation, migration, and secretion functions of SCs, ultimately improving the effect of nerve regeneration [243]. Boron ester-bonded dextran-based hydrogel patches both scavenge ROS *in vitro*, thereby protecting mitochondrial morphology and functional homeostasis in healthy RSC96 cells, and promote axonal regeneration, myelin sheath regeneration, and recovery of motor function in diabetic rats [244].

Comparing these engineered delivery platforms illustrates the diverse pathways developed to overcome the non-targeted limitations of traditional broad-spectrum antioxidants: tetrahedral framework nucleic acids (tFNAs) excel at improving drug bioavailability and enhancing SC-macrophage communication, light-responsive Chlorella hydrogels uniquely leverage biological photosynthetic oxygen generation to drive M2 macrophage polarization, and boronic ester-bonded hydrogel patches provide rapid chemical ROS scavenging to preserve SC mitochondrial homeostasis. To further enhance their therapeutic efficacy, future precision therapies must focus on integrating SC-specific targeting capabilities and developing materials that respond dynamically to both ROS and pro-inflammatory cues.

In summary, antioxidant strategies are gradually developing towards precision and targeting. The following research directions may play a positive role in the improvement of antioxidant therapeutic efficacy: (1) Design of nanoparticles with SCs targeting to achieve localized and precise delivery of therapeutic substances by combining with electrospun nerve conduits. (2) Developing ROS/pro-inflammatory factor-responsive scaffolds for precise delivery of antioxidants to damaged areas; (3) Analyzing the mechanism of interaction between SCs and the immune system at the molecular and cellular levels, and proposing ideas for targeted intervention.

## Clinical transformation and application status

Research on the explicit interplay between SCs and OS in peripheral nerve injury remains an emerging field. However, therapeutic approaches targeting antioxidant strategies and SC functional modulation are actively advancing toward clinical application. We summarize the current translational landscape (Table 5) through four key aspects: (1) Recent clinical trials, focusing on highly relevant studies registered or updated within the past 5 years; (2) Major grant support, highlighting recent projects funded by agencies like the NIH, ERC, and NSFC; (3) Seminal clinical evidence demonstrating foundational safety and efficacy; and (4) FDA-approved therapeutic drugs and nerve conduits relevant to the peripheral nervous system. In conclusion, interventions such as antioxidants, biological therapies, and tissue engineering devices all share a common mechanistic theme. They rely, to varying degrees, on either modulating the oxidative stress microenvironment or supporting the functional capacity of SCs to treat peripheral nerve injury and other neurological disorders.

**TABLE 5:** Summary of core advantages, limitations, and clinical translational timelines of various neural therapeutic strategies.

Therapeutic strategies and representative projects (Related sections)	Core advantages and translational prospects	Limitations and challenges	Current clinical status	References/Project IDs
Traditional single-lumen nerve guidance conduits (NGCs) (e.G., collagen nerve conduits, mochida nerve cuff)	Provides structural support with excellent biocompatibility. Demonstrates therapeutic efficacy comparable to nerve autografts, holding tremendous potential as an alternative therapy	Lacks the capacity for active microenvironmental modulation; exhibits suboptimal repair efficacy for long-gap nerve defects (>3 cm); some products have a relatively short shelf life	FDA-approved and commercialized; clinical data indicates favorable repair outcomes for defects <3 cm	NCT01809002; 510(k): K233322
Broad-spectrum antioxidants and Systemic drugs (e.g., NAC, ALA, SKYCLARYS)	Mitigates oxidative stress (OS) and nerve injury by activating pathways such as Nrf2; effectively improves motor function and symptoms of diabetic peripheral neuropathy	Broad-spectrum in nature, lacking specific targeting to SCs or localized injured nerves; certain drugs present adverse side effects; the precise molecular mechanisms remain incompletely elucidated	Mixed stage; SKYCLARYS is FDA-approved, while NAC and others remain in the clinical trial phase	NDA: 216718; zzNCT04481035
SC Biotherapies and genetic Pathway regulation (e.g., ahSC injection, stem cell co-transplantation, PPARγ/Netrin-1 pathways)	The introduction of SCs yields superior therapeutic outcomes compared to stem cells alone; directly targets and alleviates inflammation and OS through autophagy and specific signaling pathways	Clinical trials suffer from small sample sizes and recruitment difficulties; pathway regulation mechanisms are highly complex (e.g., mismatched expression levels may induce adverse effects), leading to prolonged bench-to-bedside translational cycles	Early clinical/Basic research stage; ahSC injection is in clinical trials (progressing slowly), while pathway regulation remains in the basic research phase	NCT05541250; NIH: 1R01DE033674-01
Biomimetic and conductive smart nerve scaffolds	Restores neural bioelectrical conduction and actively scavenges microenvironmental ROS; highly mimics ECM topological structures to guide targeted axonal regeneration	The long-term *in vivo* degradability and safety of nanomaterials (e.g., graphene) require further validation; achieving perfect dynamic coupling between material degradation rates and regeneration cycles remains challenging	Preclinical research stage	Section: “Innovative functional neural Stents”
ROS-responsive targeted delivery and engineered exosomes	Achieves precise spatiotemporal control, specifically responding to high-ROS microenvironments; exosomes possess innate high biocompatibility and targeted delivery capabilities	Response specificity and therapeutic durability under complex multiple *in vivo* stimuli remain questionable; faces significant technical bottlenecks in large-scale standardized production, purification, and cargo loading efficiency	Early preclinical/Proof-of-concept stage	Sections: “The new concept of intelligent drug delivery system” and “Precision antioxidant therapy based on OS-SCs interaction mechanism”
4D Bioprinting technology	Endows scaffolds with the capacity for time-dependent adaptive morphological changes; allows for precise programming of spatiotemporal gradient release of neurotrophic factors and antioxidants	Currently in the initial exploratory phase; involves extreme technical difficulty and struggles to perfectly integrate and respond to complex multimodal physiological signals post-implantation	Proof-of-concept/*In vitro* research stage	Section: “4D bioprinting technology”

Abbreviations: NGCs, Nerve Guidance Conduits; NAC, N-acetylcysteine; ALA, Alpha-lipoic acid; OS, Oxidative Stress; SCs, Schwann Cells; ahSC, Autologous human Schwann cell; ECM, Extracellular Matrix; ROS, Reactive Oxygen Species; PPARγ, Peroxisome proliferator-activated receptor gamma. NCT, National Clinical Trial; 510(k), Premarket Notification; NDA, New Drug Application; FDA, Food and Drug Administration; NIH, National Institutes of Health; NSFC, National Natural Science Foundation of China.

However, most of these strategies act indirectly on the SC-OS axis, and there are still no clinical experiments that directly target and regulate the interaction between the two. Particularly in the field of neural tissue engineering, most currently FDA-approved NGCs (such as NeuraGen®, NeuroMatrix™, Neurolac®, Neuroflex™, Reaxon®, Nerbridge®, and Avance®) are predominantly single-lumen, hollow conduits. Clinical data show that although some products perform well for short nerve defects (<3 cm), they cannot fulfill the complex biophysical and biochemical requirements for comprehensive nerve regeneration in a hostile oxidative microenvironment [245]. This suboptimal efficacy in long-gap repairs is largely attributed to the lack of an internal biomimetic ECM topographic structure, which prevents SCs from migrating and forming vital Bands of Büngner. Furthermore, the massive burst of ROS triggered by ischemia-reperfusion injury during surgical implantation often transforms these passive conduits into a “hostile zone” that accelerates SC senescence and apoptosis. Although NGCs designed for long-gap nerve defects have not yet entered clinical practice, preclinical studies have successfully achieved the regeneration of 5-cm axonal gaps and the restoration of electrophysiological functions in porcine models [246].

Translating the existing relevant designs from the preclinical stage to the clinical trial stage is currently the main challenge and opportunity. In the near-to-medium term, given their manufacturing maturity, targeted antioxidant pharmacotherapies and biomimetic/conductive nerve scaffolds are well-positioned to advance into clinical trials. By contrast, complex intelligent systems, including ROS-responsive hydrogels, engineered exosomes, and 4D bioprinting, face substantial barriers in long-term stability, dynamic signal integration, and scalable production, rendering their widespread clinical adoption a prolonged endeavor.

In the future, it is necessary to further deepen the understanding of the mechanism of interaction between OS and SCs from the molecular level to the cellular level and even the tissue structure, and actively develop intelligent materials, catheters and scaffolds that can respond in real time to the dynamic changes of ROS around SCs, to achieve precise regulation of the state and repair function of SCs.

## Discussion

This review has provided a comprehensive overview of the mechanisms linking OS and SCs in PNI, alongside emerging tissue engineering strategies. Several limitations of this study merit consideration. Only English language literature was included, potentially omitting relevant studies in other languages, and the search cutoff of June 2026 excludes more recent findings. By concentrating on SCs, the review downplayed the contributions of other cells such as macrophages and neutrophils, which simplifies the actual microenvironmental interaction network. Furthermore, the conclusions apply primarily to OS evolution and SC stress repair in PNI and do not extensively address metabolic or genetic peripheral neuropathies; mechanistic extrapolation beyond this context requires caution.

Despite these constraints, the evidence highlights that OS driven microenvironmental disorder poses a severe challenge to nerve repair. ROS play a dual role: excessive ROS damage lipids, proteins, and DNA, triggering neuronal and SC injury and inflammatory cascades, while physiological levels support neural repair through immune defense and intracellular signaling. Importantly, even when ROS mediated effects appear similar across organs, tissue specific differences must be respected, and findings from other tissues cannot be directly transferred to peripheral nerve without proper validation. Existing research has centered on diabetic and neurodegenerative neuropathies, leaving the ROS related mechanisms in mechanical nerve trauma insufficiently explored. Future studies should therefore employ well established PNI models to systematically delineate the molecular pathways governing ROS driven degeneration and repair.

ROS imbalance drives SCs toward a senescent phenotype, which critically deteriorates the regenerative microenvironment and limits axonal regeneration. Traditional neural scaffolds, including FDA approved conduits based on collagen or polyglycolic acid, primarily offer passive structural support and lack the capacity to actively neutralize excessive ROS. Elucidating the spatiotemporal distribution of ROS, their damaging effects on SCs, and the regulation of antioxidant pathways is thus essential for creating smart responsive scaffolds with integrated anti-inflammatory and antioxidant capabilities. Recent biomanufacturing advances are shifting neural tissue engineering from structural biomimicry to functional regeneration. Next-generation nerve guidance conduits can overcome autograft limitations by combining ROS modulating mechanisms with dynamic drug delivery, enabling precise redox homeostasis control while promoting vascular regeneration and neural electrical network reconstruction.

A multi strategy integrated conduit design is highly valuable in the pro-oxidant injury environment. ROS responsive materials can trigger localized drug release or structural changes in reaction to fluctuating ROS levels. Engineered exosomes with surface or cargo modifications further enhance biological targeting, and pre programmed four dimensional structures improve adaptability to microenvironmental changes. Together, these approaches yield a tridimensional synergy of response, targeting, and adaptation centered on the ROS axis. Clinical translation remains the core challenge. Although personalized conduits improve anatomical matching, their high cost restricts large scale application; developing tiered, cost effective, and standardized solutions for short and long gap injuries is therefore necessary. Moreover, the abundance of material choices has resulted in a lack of unified evaluation standards. Even mainstream materials such as chitosan and polycaprolactone lack consensus benchmarks for degradation rate, biocompatibility, and mechanical properties, which confines most active therapies to the laboratory stage.

Future research should concentrate on the following core directions: (1) Emphasizing the timing and spatial distribution of pathogenic ROS through highly sensitive monitoring technologies with advanced spatiotemporal resolution. (2) Overcoming the limitations of traditional, single-function scaffolds (including passive FDA-approved conduits) by developing multimodal intelligent scaffolds that integrate antioxidant capacity, electrical conductivity, and immune modulation. (3) Further elucidating the bidirectional interplay between mitochondrial autophagy–mediated ROS clearance and the broader OS microenvironment. (4) Promoting the visualization of peripheral nerve regeneration status during multidimensional regulatory processes. (5) Actively advancing rigorous preclinical and clinical trials. This includes establishing cross-laboratory consensus standards for material properties (such as degradation and mechanical compliance) and utilizing large animal models for long-gap (>5 cm) nerve defects to bridge the vast gap between preclinical success and actual clinical efficacy.
